# Immune gene prognostic signature for disease free survival of gastric cancer: Translational research of an artificial intelligence survival predictive system

**DOI:** 10.1016/j.csbj.2021.04.025

**Published:** 2021-04-12

**Authors:** Zhiqiao Zhang, Tingshan He, Liwen Huang, Jing Li, Peng Wang

**Affiliations:** Department of Infectious Diseases, Shunde Hospital, Southern Medical University, Shunde, Guangdong, China

**Keywords:** GC, gastric cancer, TCGA, The Cancer Genome Atlas, GEO, the Gene Expression Omnibus, ROC, receiver operating characteristic, DFS, disease free survival, HR, hazard ratio, CI, confidence interval, AJCC, the American Joint Committee on Cancer, SD, standard deviation, DCA, decision curve analysis, Immune gene, Transcription factor, Gastric cancer, Disease free survival, Prognostic signature

## Abstract

The progress of artificial intelligence algorithms and massive data provide new ideas and choices for individual mortality risk prediction for cancer patients. The current research focused on depict immune gene related regulatory network and develop an artificial intelligence survival predictive system for disease free survival of gastric cancer.

Multi-task logistic regression algorithm, Cox survival regression algorithm, and Random survival forest algorithm were used to develop the artificial intelligence survival predictive system.

Nineteen transcription factors and seventy immune genes were identified to construct a transcription factor regulatory network of immune genes. Multivariate Cox regression identified fourteen immune genes as prognostic markers. These immune genes were used to construct a prognostic signature for gastric cancer. Concordance indexes were 0.800, 0.809, and 0.856 for 1-, 3- and 5- year survival. An interesting artificial intelligence survival predictive system was developed based on three artificial intelligence algorithms for gastric cancer. Gastric cancer patients with high risk score have poor survival than patients with low risk score.

The current study constructed a transcription factor regulatory network and developed two artificial intelligence survival prediction tools for disease free survival of gastric cancer patients. These artificial intelligence survival prediction tools are helpful for individualized treatment decision.

## Graphical abstract

**Figure:**
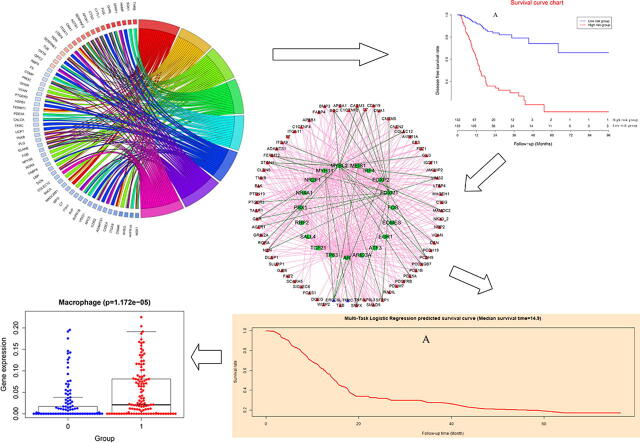

## 1 Background

Epidemiological data demonstrated gastric cancer (GC) is one of the leading digestive malignant tumors and ranks second for tumor-related deaths with 782,685 deaths in 2018 [1]. Although advances in early screening, diagnosis, and treatments reduced mortality to some extent [2], [3], the prognosis of gastric cancer patients were still unsatisfactory [4]. From a clinical point of view, early identification of high risk GC patients with high mortality and more precise individualized treatments are helpful to improve the prognosis of high risk GC patients. Therefore, reliable and precise individual mortality risk prediction is of great significance for optimizing individual treatment effect.

Great progress has been made in precision medicine in recent years [5], [6]. Precision medical predictive tools can be used in predicting individual mortality risk in different time-points and the efficacy for different treatments. [7], [8], [9]. However, precision medical predictive tools for predicting mortality risk of gastric cancer patients have not been able to meet the needs of individualized treatment.

Bioinformatics advances provided tremendous impetus to precision medical research in tumorigenesis and progression. Bioinformatics is helpful to explore the intrinsic biological regulatory mechanisms and potential pathways for tumorigenesis and progression [10], [11], [12], [13]. In recent years, more and more studies have focused on the important role of immune microenvironment in tumorigenesis and progression [14], [15]. Jiang et al. developed a prognostic signature in predicting the prognosis of gastric cancer patients [16]. Yang et al. developed a prognostic signature based on immune genes to predict overall survival of GC patients [17]. However, this prognostic signature did not provide calculation formula and was limited for clinical application. Therefore, it is valuable to develop individualized precision medical predictive tools for early identification of gastric cancer with high mortality risk.

Precision medical predictive tools can provide individualized mortality risk prediction and help clinicians early identify patients with high mortality risk. Recently, our team has successfully developed several precision medical predictive tools based on genetic data for different tumors [18], [19], [20]. In recent years, the development of artificial intelligence algorithms provides more choices for the predictive studies of tumor prognosis. Multi-task logistic regression algorithm, Cox survival regression algorithm, and random survival forest algorithm have been used to improve the accuracy of predictive models and prognostic models [21], [22], [23], [24], [25], [26], [27], [28], [29], [30], [31], [32], [33], [34]. Therefore the current research was devoted to explore potential immune regulatory mechanism for prognosis of GC and construct artificial intelligence survival prediction tools for predicting individual mortality risk in different time-points.

## 2 Methods

### 2.1 Study datasets

Model dataset was downloaded from TCGA database, involving 22,412 mRNAs from 375 GC specimens and 32 normal specimens (TCGA, PanCancer Atlas, Cell 2018, http://www.cbioportal.org/study/summary?id=stad_tcga_pan_can_atlas_2018). Two hundred and sixty five GC patients were included after moving patients with follow-up information less than one month. Validation dataset (GSE62254) was obtained from GEO database. GSE62254 dataset contained two hundred and seventy nine patients and 19,765 mRNAs (GPL570 platform). Probe IDs were translated to official gene symbols according to Gencode.v29 background file.

### 2.2 Differentially expressed analyses

Differentially expressed analyses between GC samples and normal samples were performed by R package “edgeR” [35]. Normalization of original data was performed by Trimmed mean of M values (TMM) method. *P* value < 0.05 and log_2_ |fold change| >1 were set as cut off values for differentially expressed analyses.

### 2.3 Immune gene and transcription factor

Immune genes were obtained from Immunology Database and Analysis Portal (ImmPort) database [36]. Transcription factors act an important role in molecular biology regulation mechanisms of tumorigenesis and progression. To explore potential regulatory relationships between transcription factors and immune genes, three hundred and eighteen transcription factors were identified from Cistrome Cancer database [37]. Associations between tumor infiltrating immune cells and immune genes were explored via Tumor IMmune Estimation Resource (TIMER) database (https://cistrome.shinyapps.io/timer/) [37]. The tumor infiltrating immune cell dataset was downloaded from Tumor IMmune Estimation Resource database involved 11,509 TCGA samples and original values of six tumor infiltrating immune cells (B_cell, CD4_Tcell, CD8_Tcell, Neutrophil, Macrophage, and Dendritic).

### 2.4 Statistical analyses and artificial intelligence algorithms

Statistical analyses were carried out by using SPSS Statistics 19.0 (SPSS Inc.,USA). Artificial intelligence algorithms were performed by Python language 3.7.2 and R software 3.5.2 (https://www.r-project.org/) in previous studies [27], [28], [29], [30], [31], [32], [33], [34]. Artificial intelligence algorithms were carried out according to the original articles: Multi-task logistic regression [23], [38], Cox survival regression [24], and Random survival forest [21], [22]. *P* value < 0.05 was considered statistically significant.

## 3 Results

### 3.1 Study datasets

Flow chart in current study was presented in Supplementary Fig. 1. Model cohort contained 265 GC patients and validation cohort contained 279 GC patients. The comparisons of clinical parameters between model cohort and validation cohort were presented in Table 1.

**Table 1:** The clinical features of patients in model cohort and validation cohort.

	TCGA cohort	GSE62254 cohort	*P* value
Patient number	265	279	
Death [n(%)]	98(37.0)	152(54.5)	<0.001
Survival time for living patients(mean ± SD, month)	19.6(12.3,32.2)	60.0(49.0,76.0)	<0.001
Survival time for dead patients (mean ± SD, month)	10.6(6.0,15.4)	9.4(3.9,18.1)	0.827
Age (mean ± SD, year)	64.4 ± 10.6	61.9 ± 11.3	0.008
Male [(n)%]	175(66.0)	181(94.9)	0.776
AJCC Stage (IV)	23	75	<0.001
AJCC Stage (III)	103	86	
AJCC Stage (II)	91	89	
AJCC Stage (I)	41	29	
AJCC Stage (NA)	7	0	
AJCC PT (T4)	66	20	<0.001
AJCC PT (T3)	121	84	
AJCC PT (T2)	62	175	
AJCC PT (T1)	16	0	
AJCC PT (NA)	0	0	
AJCC PN (N3)	53	50	0.006
AJCC PN (N2)	55	73	
AJCC PN (N1)	67	119	
AJCC PN (N0)	88	37	
AJCC PN (NA)	2	0	
AJCC PM (M1)	22	27	0.578
AJCC PM (M0)	243	252	
AJCC PM (NA)	0	0	
Targeted molecular therapy (Yes)	78	NA	
Targeted molecular therapy (No)	79	NA	
Targeted molecular therapy (NA)	108	NA	
Radiation treatment adjuvant (Yes)	0	NA	
Radiation treatment adjuvant (No)	155	NA	
Radiation treatment adjuvant (NA)	110	NA	
Barretts esophagus (Yes)	12	NA	
Barretts esophagus (No)	151	NA	
Barretts esophagus (NA)	102	NA	
H pylori infection (Yes/No/NA)	15	NA	
H pylori infection (No)	117	NA	
H pylori infection (NA)	133	NA	

*Note*: NA, missing data; SD: standard deviation; AJCC: American Joint Committee on Cancer.

### 3.2 Differentially expressed analyses

**Fig. 1:**
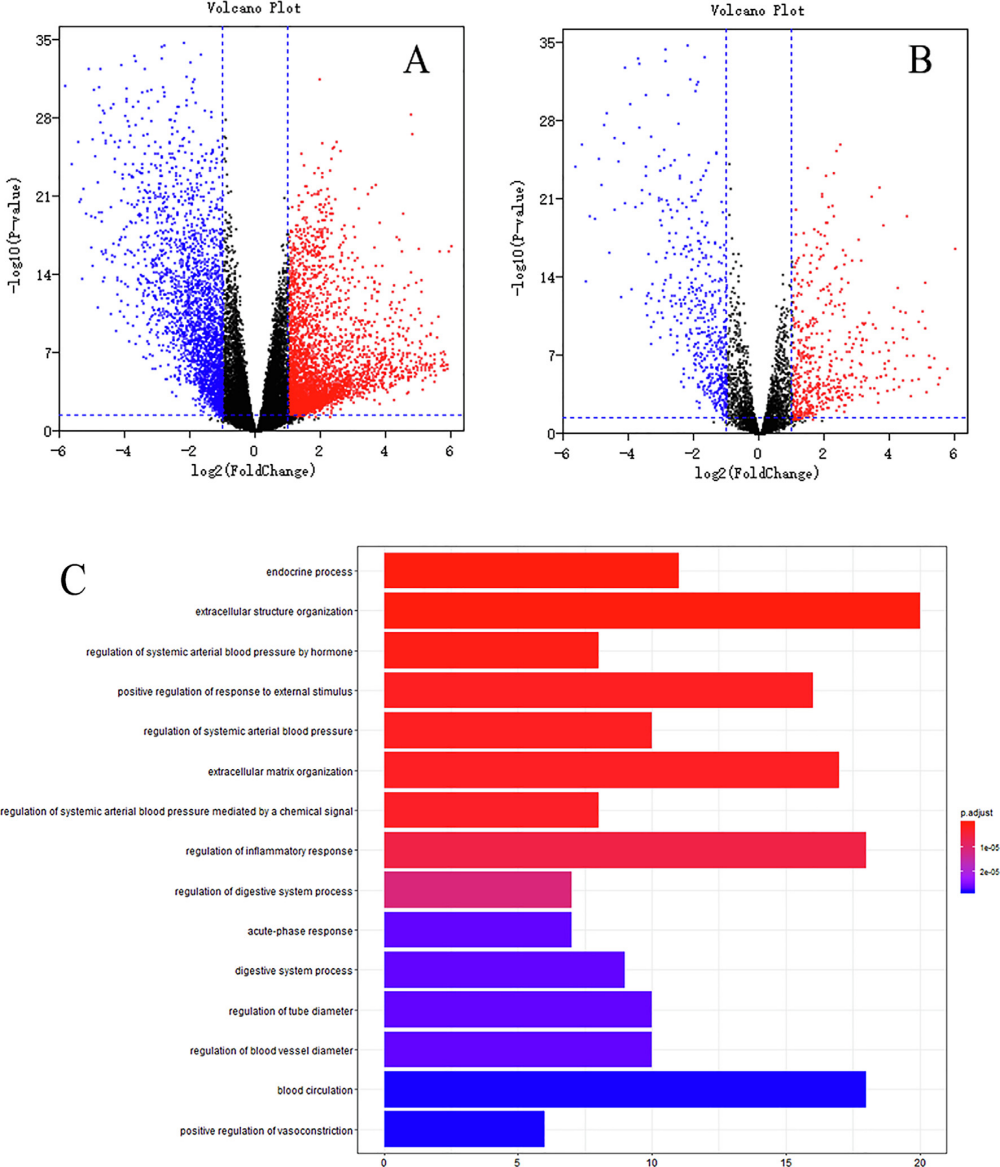
Differentially expression and gene functional.

Differentially expressed analyses (Fig. 1A) identified 6047 differentially expressed mRNAs (3539 up-regulated and 2508 down-regulated) out of 22,412 mRNAs. There were 3691 immune genes after interaction between mRNA symbols and immune genes from ImmPort database. Differentially expressed analyses (Fig. 1B) identified 2352 differentially expressed immune genes (1235 up-regulated and 1117 down-regulated).

### 3.3 Functional enrichment analyses

Potential biological functions of immune genes were explored through Gene Ontology (GO) functional enrichment analyses. Bar plot (Fig. 1C), bubble plot (Supplementary Fig. 2) and chord plot (Supplementary Fig. 3) indicated potential biological functions of immune genes as following: collagen-containing extracellular matrix, extracellular matrix, endocrine process, extracellular structure organization, regulation of systemic arterial blood pressure by hormone, regulation of systemic arterial blood pressure, positive regulation of response to external stimulus, extracellular matrix organization, regulation of systemic arterial blood pressure mediated by a chemical signal, platelet alpha granule lumen, platelet alpha granule, regulation of inflammatory response, regulation of digestive system process, secretory granule lumen, and cytoplasmic vesicle lumen.

### 3.4 Prognostic immune genes and regulatory network

**Fig. 2:**
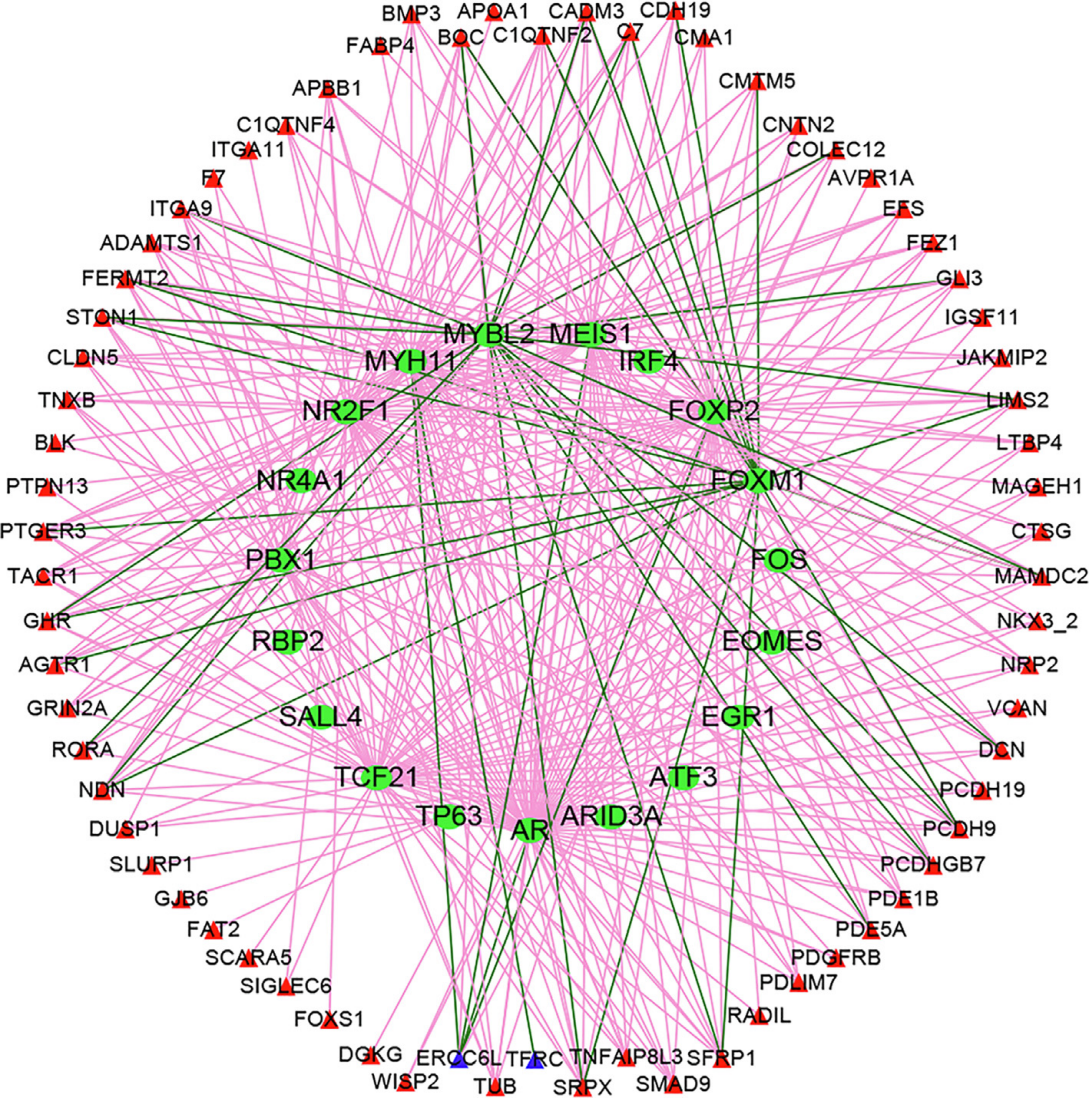
Immune gene regulatory network chart.

There were 160 immune genes identified as prognostic markers for GC via univariate Cox regression. Transcription factor is a key link in the molecular regulatory pathway. To better understand the regulatory relationship between transcription factors and immune genes, the current study performed correlation analyses to identify transcription factors closely related to immune genes. According to cut off values of |correlation coefficient| > 0.5 and *P* value < 0.01, 19 transcription factors and 70 immune genes were identified to construct a transcription factor regulatory network of immune genes (Fig. 2) via Cytoscape v3.6.1 [39].

### 3.5 Construction of prognostic signature

**Fig. 3:**
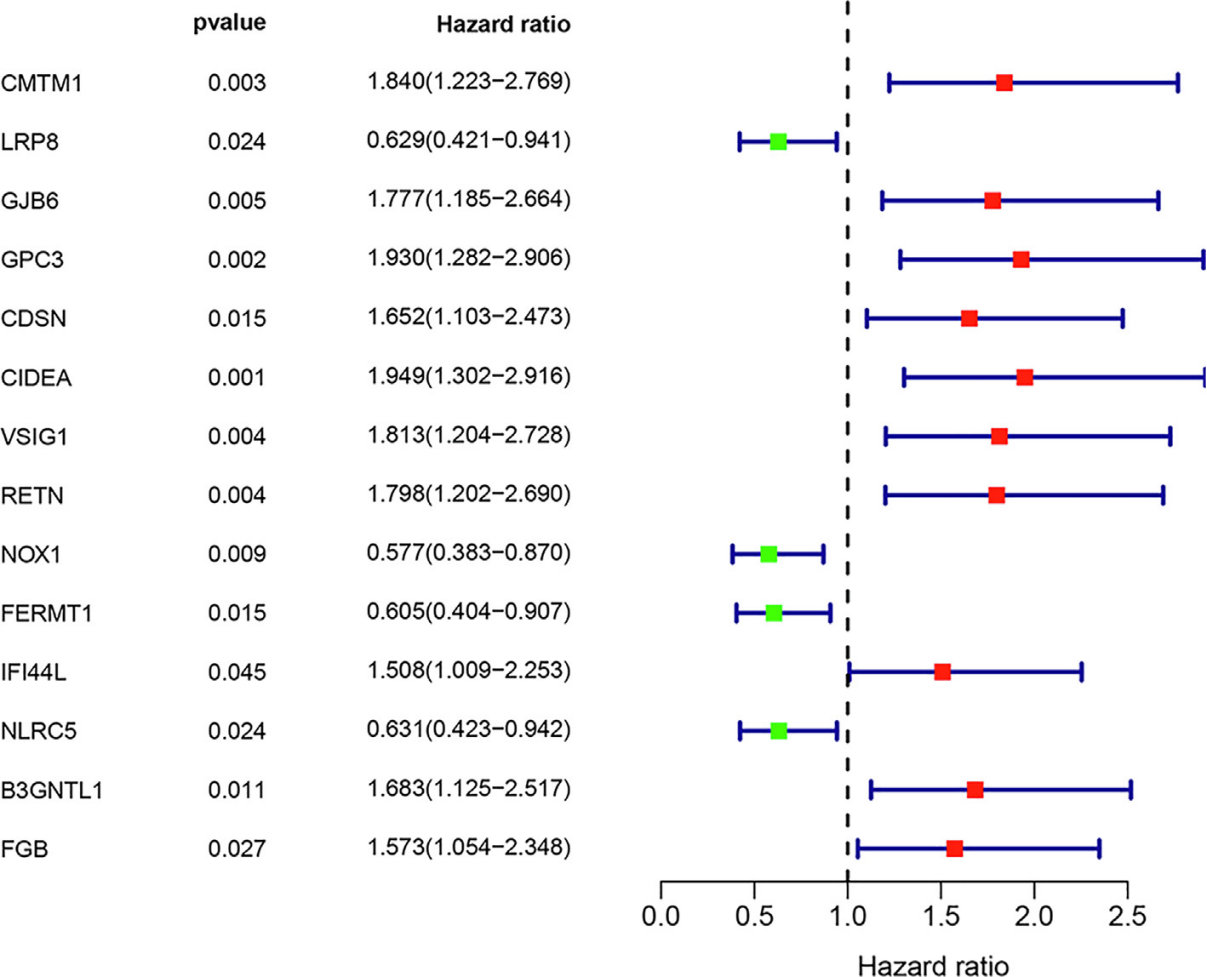
Immune gene survival forest chart.

**Fig. 4:**
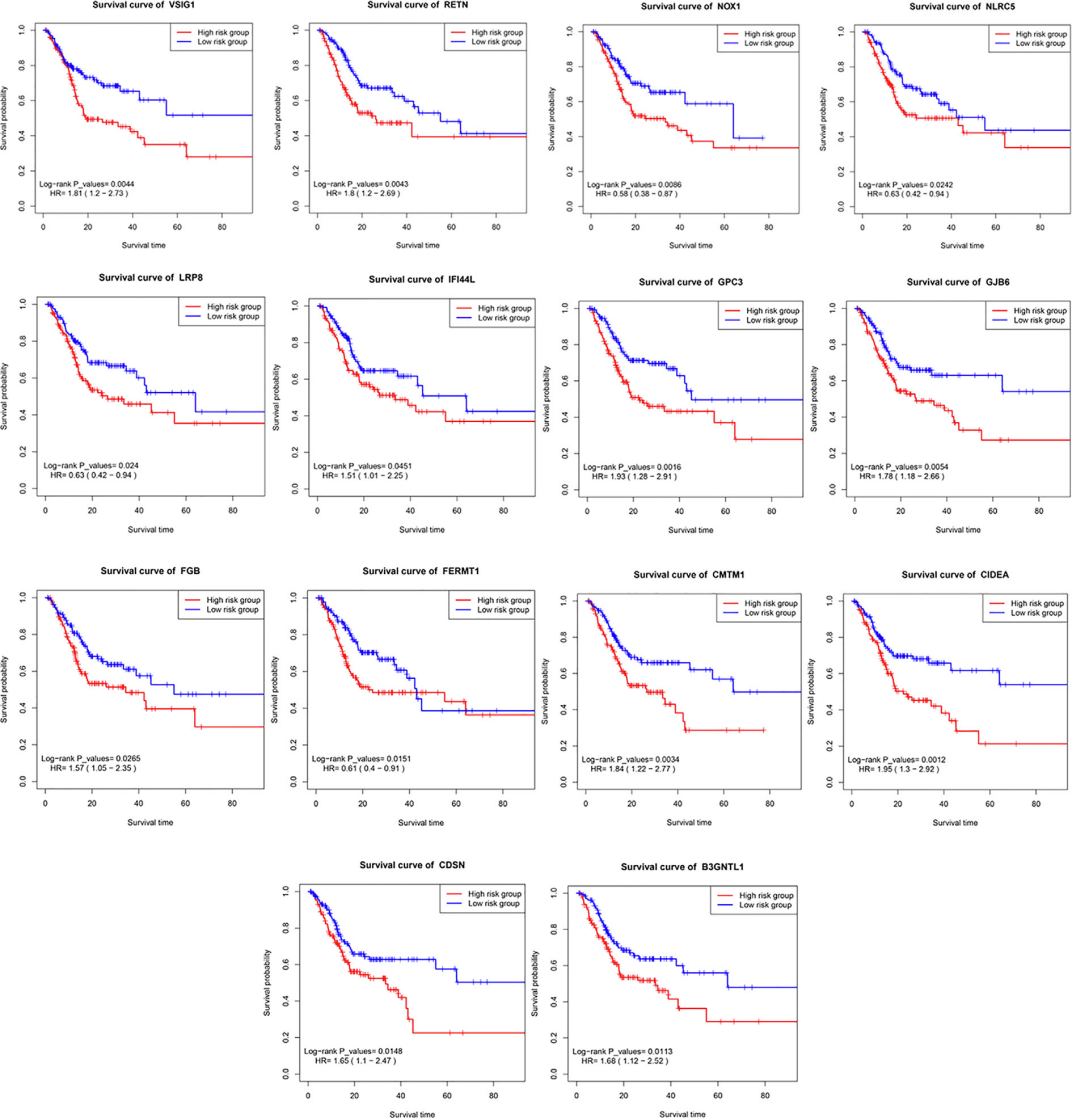
Survival curves of immune genes.

**Fig. 5:**
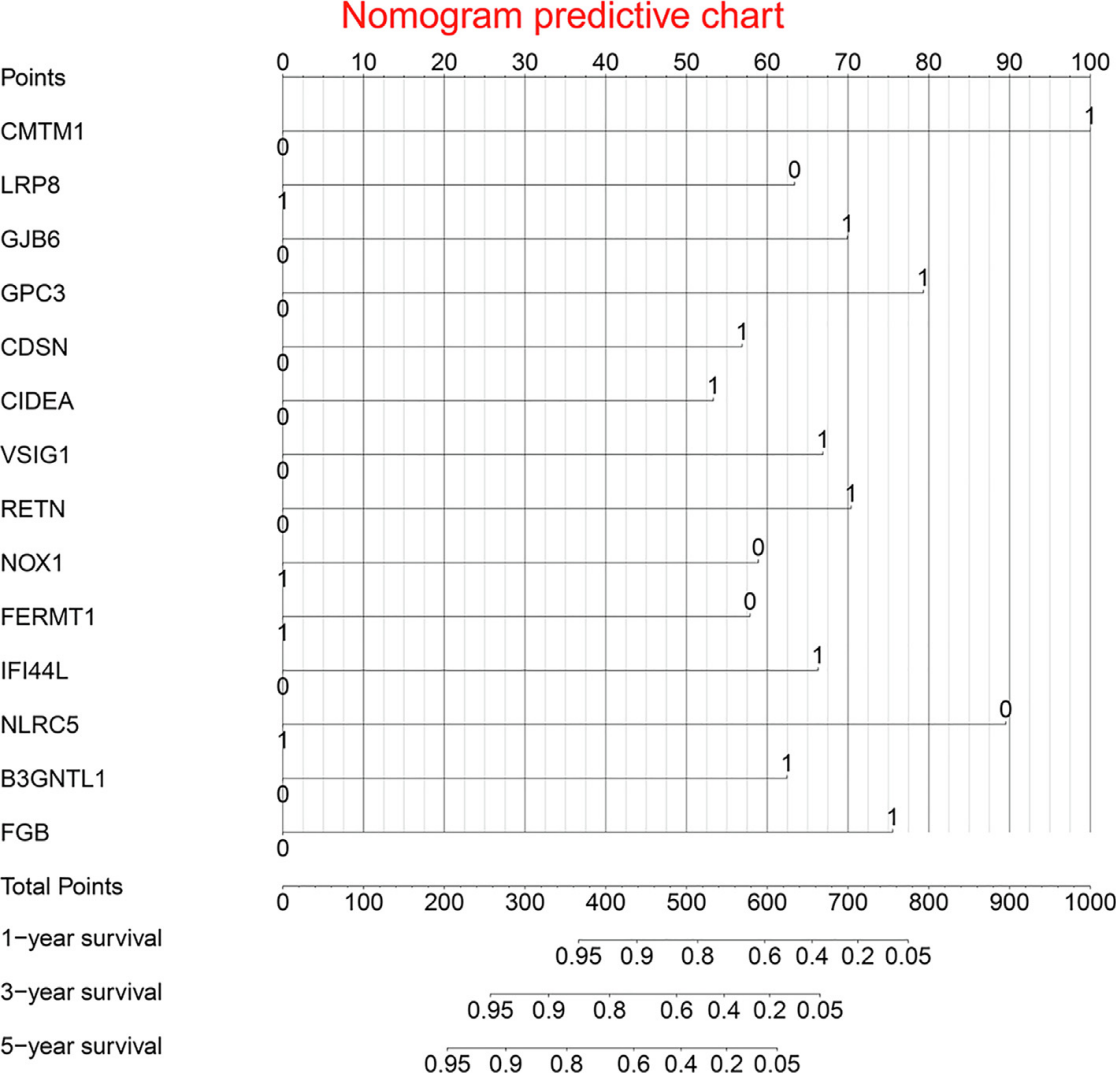
Prognostic nomogram chart.

Out of previous prognostic immune genes, fourteen immune genes were identified as independent risk factors for DFS (Table2). The risk factor forest chart and survival curve chart of fourteen immune immune genes were presented in Figs. 3 and 4. The prognostic signature was calculated with the following formula: The risk score = (0.5307*CIDEA) + (0.665*VSIG1) + (0.6206*B3GNTL1) + (-0.5741*FERMT1) + (0.7004*RETN) + (-0.8919*NLRC5) + (0.6959*GJB6) + (0.7907*GPC3) + (0.9957*CMTM1) + (0.6608*IFI44L) + (-0.6322*LRP8) + (0.7525*FGB) + (-0.5867*NOX1) + (0.5657*CDSN). A prognostic nomogram was presented in Fig. 5.

**Table 2:** Information of prognostic genes.

	Univariate analysis			Multivariate analysis			
Gene	HR	95% CI	*P*-value	coefficient	HR	95% CI	*P*-value
CIDEA(High/Low)	1.949	1.302-2.916	0.001	0.531	1.700	1.104-2.620	0.016
VSIG1(High/Low)	1.813	1.204-2.728	0.004	0.665	1.945	1.242-3.044	0.004
B3GNTL1(High/Low)	1.683	1.125-2.517	0.011	0.621	1.860	1.122-3.084	0.016
FERMT1(High/Low)	0.605	0.404-0.907	0.015	−0.574	0.563	0.343-0.925	0.023
RETN(High/Low)	1.798	1.202-2.690	0.004	0.700	2.015	1.267-3.205	0.003
NLRC5(High/Low)	0.631	0.423-0.942	0.024	−0.892	0.410	0.253-0.664	0.000
GJB6(High/Low)	1.777	1.185-2.664	0.005	0.696	2.006	1.297-3.102	0.002
GPC3(High/Low)	1.930	1.282-2.906	0.002	0.791	2.205	1.409-3.450	0.001
CMTM1(High/Low)	1.840	1.223-2.769	0.003	0.996	2.707	1.537-4.765	0.001
IFI44L(High/Low)	1.508	1.009-2.253	0.045	0.661	1.936	1.214-3.088	0.006
LRP8(High/Low)	0.629	0.421-0.941	0.024	−0.632	0.531	0.330-0.856	0.009
FGB(High/Low)	1.573	1.054-2.348	0.027	0.753	2.122	1.340-3.362	0.001
NOX1(High/Low)	0.577	0.383-0.870	0.009	−0.587	0.556	0.348-0.888	0.014
CDSN(High/Low)	1.652	1.103-2.473	0.015	0.566	1.761	1.108-2.798	0.017

*Note*: HR, hazard ratio; CI, confidence interval. The medians of gene expression values were used as cut-off values to stratify gene expression values into high expression group (as value 1) and low expression group (as value 0).

Survival curve analyses of immune genes (Fig. 3) demonstrated that DFS were significantly different between different immune expression status (*P* < 0.05). The predictive value distribution chart and survival status scatter plot were presented in Supplementary Figs. 4 and 5.

### 3.6 Predictive performance in model cohort

**Fig. 6:**
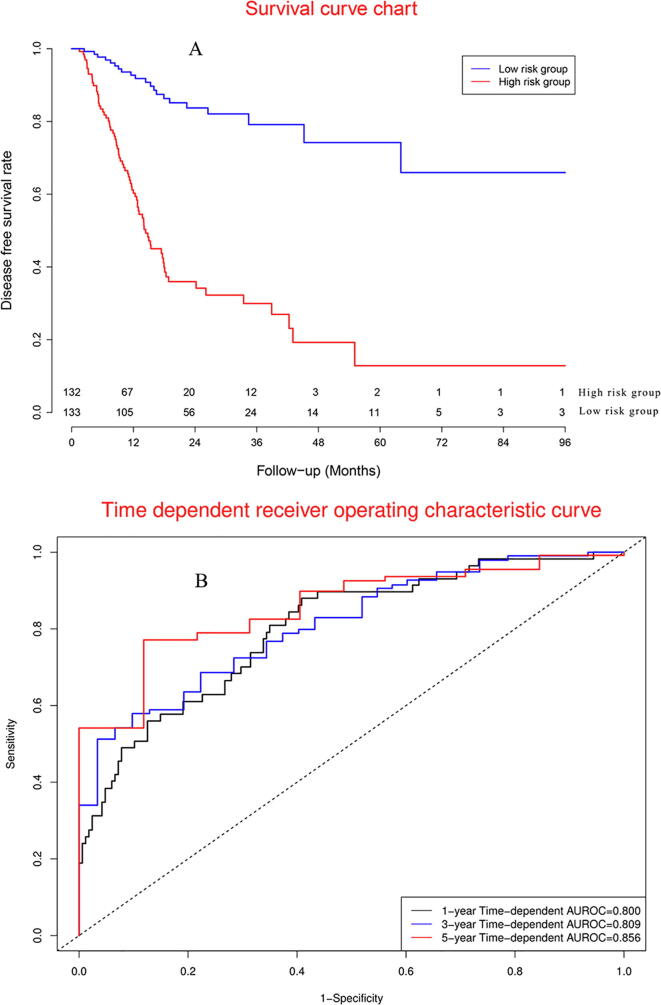
Clinical performance in model cohort.

According to median of prognostic signature score, Fig. 6A demonstrated that there was significant difference between two groups. Concordance indexes were 0.800, 0.809, and 0.856 for 1-year, 3-year, and 5-year DFS (Fig. 6B). Calibration curves were showed in Supplementary Fig. 6.

### 3.7 Predictive performance in validation cohort

**Fig. 7:**
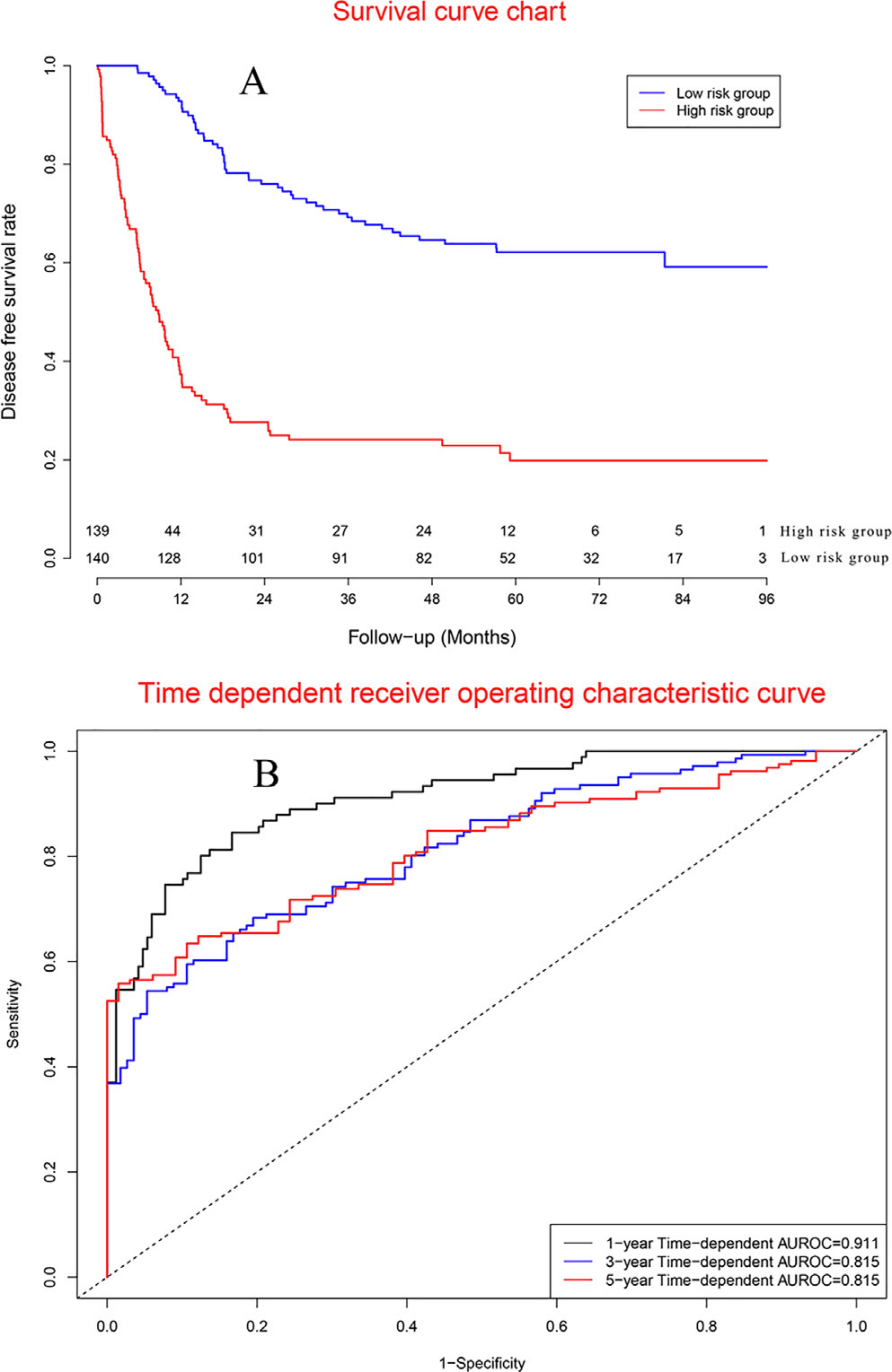
Clinical performance in validation cohort.

Survival curves (Fig. 7A) demonstrated that DFS in high risk group was significantly poor than that in low risk group. Concordance indexes were 0.911, 0.815, and 0.815 for 1-year, 3-year, and 5-year DFS (Fig. 7B). Calibration curves were showed in Supplementary Fig. 7. Decision curve charts were presented in Supplementary Fig. 8.

### 3.8 Artificial intelligence survival predictive system

**Fig. 8:**
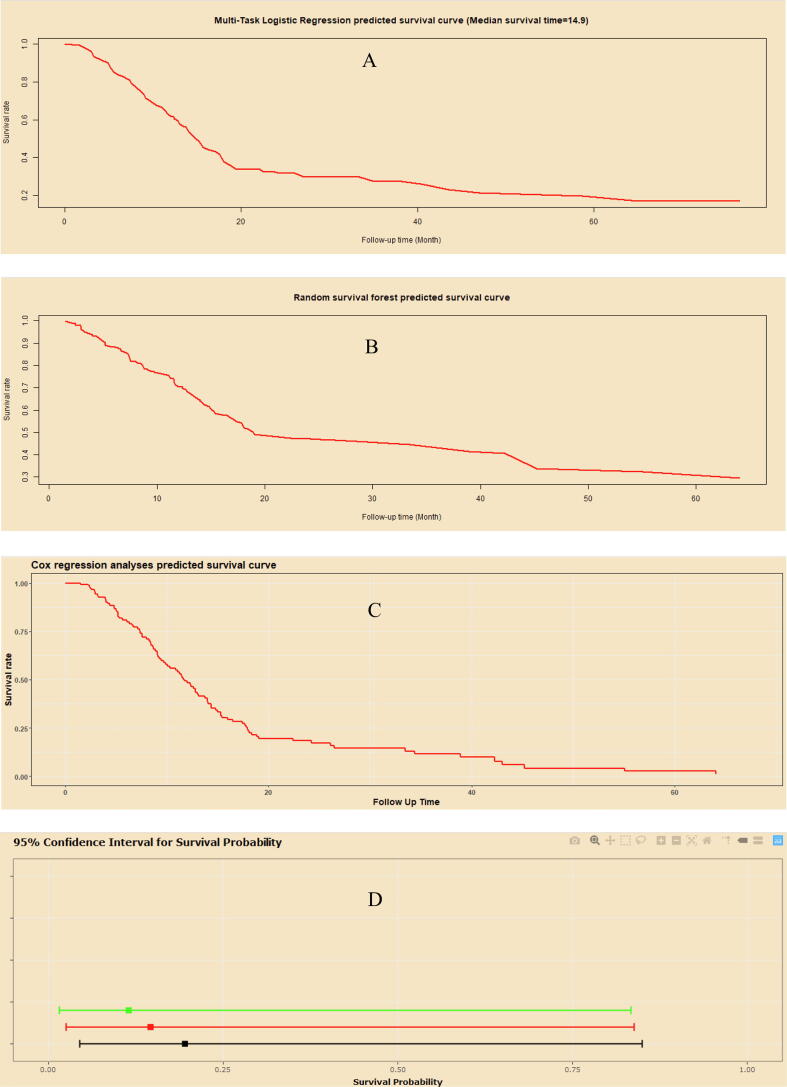
Home page of artificial algorithm survival predictive system.

An artificial intelligence survival predictive system was developed to provide on-line prediction for DFS (Fig. 8). This artificial intelligence survival predictive system was provided at: https://zhangzhiqiao7.shinyapps.io/Smart_Cancer_Survival_Predictive_System_15_GC_D1006/. Three individual mortality risk predictive curves predicted by, Multi-task logistic regression (MTLR) algorithm (Fig. 8A), Random survival forest (RFS) algorithm (Fig. 8B), and Cox survival regression algorithm (Fig. 8C). This artificial intelligence survival predictive system could provide 95% confidence interval of predicted mortality and median survival time for an individual patient.

### 3.9 Gene survival analysis screen system

**Fig. 9:**
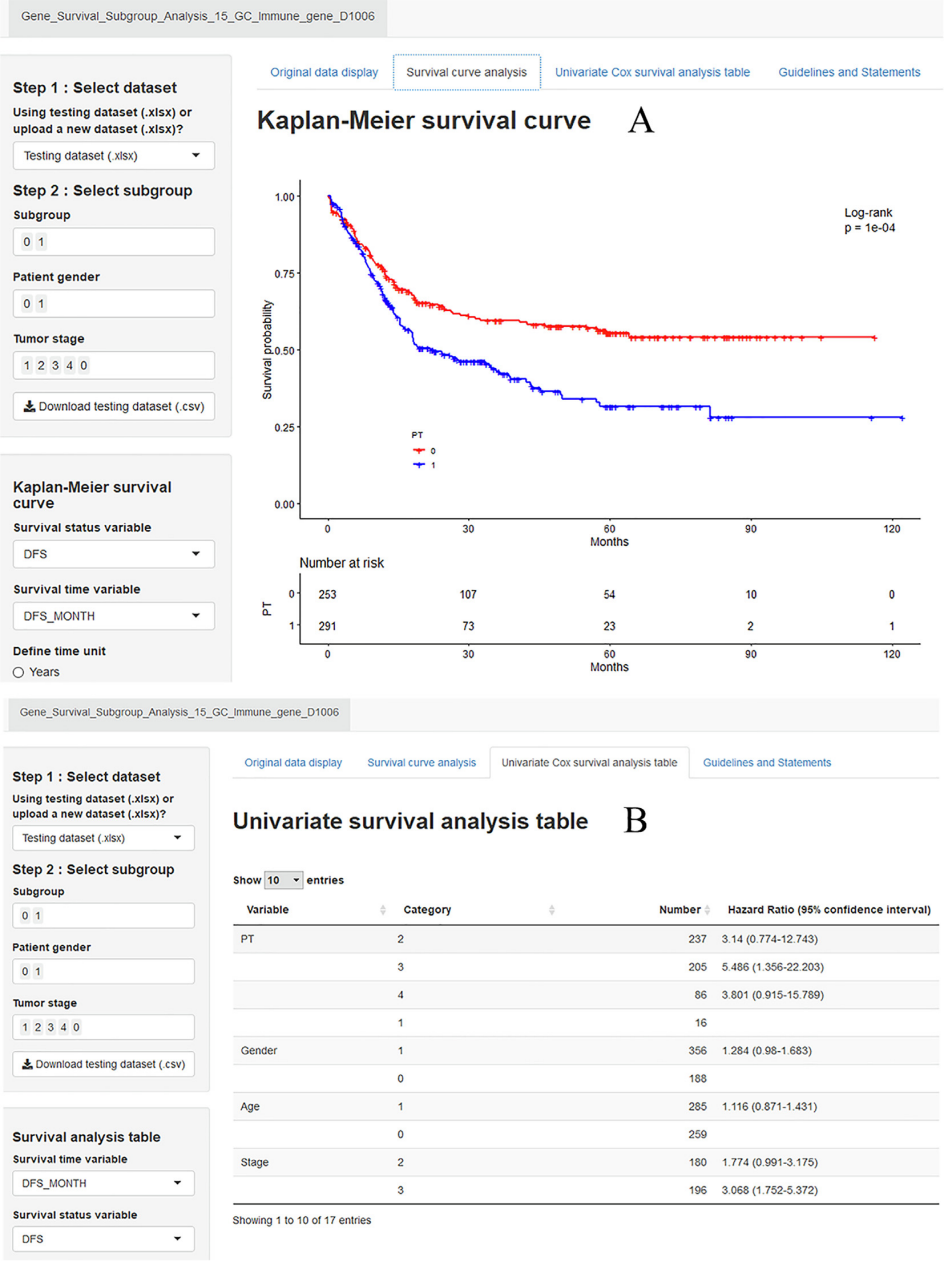
Home page of Gene Survival Analysis Screen System.

Univariate Cox regression recognized 160 immune genes as prognostic markers for GC. A precision medical predictive tool named Gene Survival Analysis Screen System was developed to explore the prognostic influence of these 160 immune genes in different subgroups (Fig. 9). Gene Survival Analysis Screen System was provided at: https://zhangzhiqiao7.shinyapps.io/Gene_Survival_Subgroup_Analysis_15_GC_D1006/.

### 3.10 Independence assessment

In model cohort, this prognostic signature was an independent risk factor for DFS (Table 3). In validation cohort, prognostic signature, American Joint Committee on Cancer PM, and gender were independent risk factors for DFS.

**Table 3:** Results of Cox regression analyses.

	Univariate analysis			Multivariate analysis			
	HR	95% CI	*P*-value	Coefficient	HR	95% CI	*P*-value
TCGA cohort (n = 265)							
Age(High/Low)	0.858	0.577-1.274	0.447	0.265	1.303	0.854-1.988	0.220
Gender (Male/Female)	1.607	1.072-2.409	0.022	0.114	1.120	0.610-2.058	0.714
AJCC PT (T3-4/T1-2)	1.385	0.876-2.191	0.164	0.424	1.527	0.885-2.635	0.128
AJCC PN (N2-3/N0-1)	1.626	1.094-2.417	0.016	0.260	1.296	0.753-2.233	0.349
AJCC PM (M1/M0)	1.056	0.512-2.178	0.883	−0.098	0.907	0.435-1.891	0.794
AJCC stage (IV,III/II,I)	1.872	1.173-2.988	0.009	0.409	1.505	0.936-2.421	0.092
Prognostic model (High/Low)	6.011	3.685-9.807	<0.001	1.809	6.103	3.693-10.080	<0.001
GSE62254 cohort (n = 279)							
Age(High/Low)	1.347	0.979-1.853	0.068	0.257	1.292	0.935-1.786	0.120
Gender (Male/Female)	3.546	2.425-5.183	<0.001	0.865	2.376	1.268-4.453	0.007
AJCC PT (T3-4/T1-2)	2.270	1.649-3.125	<0.001	0.188	1.207	0.809-1.801	0.356
AJCC PN (N2-3/N0-1)	2.848	2.051-3.956	<0.001	0.074	1.077	0.668-1.735	0.762
AJCC PM (M1/M0)	3.501	2.265-5.413	<0.001	0.847	2.332	1.471-3.696	<0.001
AJCC stage (IV,III/II,I)	1.033	0.739-1.445	0.848	0.242	1.273	0.902-1.798	0.170
Prognostic model (High/Low)	4.294	3.050-6.044	<0.001	1.293	3.645	2.542-5.228	<0.001

*Note*: AJCC, the American Joint Committee on Cancer; HR, hazard ratio; CI, confidence interval. The median of Prognostic model scores was used as the cut-off value to stratify gastric cancer patients into high risk group and low risk group.

### 3.11 Subgroup analyses

**Fig. 10:**
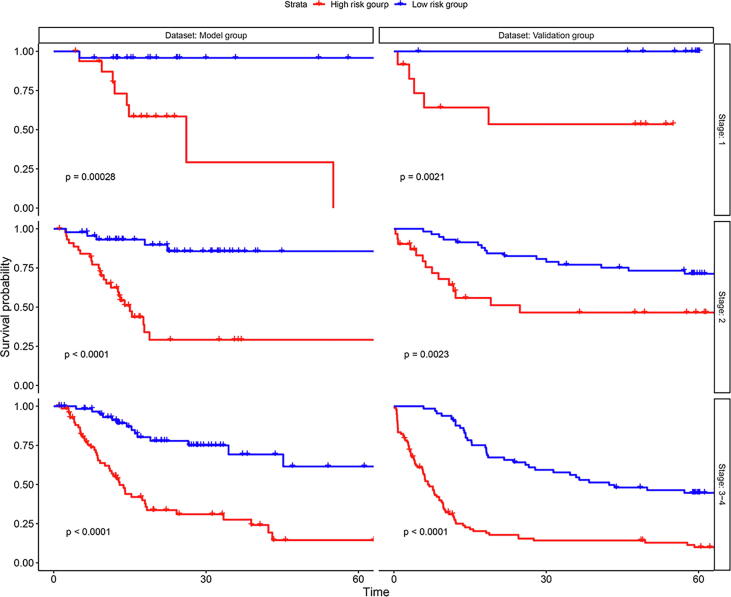
Survival curve subgroup analysis.

Subgroup analyses demonstrated that DFS in high risk group was significantly poor than that in low risk group for different stage groups in both model cohort and validation cohort (Fig. 10).

### 3.12 Clinical correlation analyses

**Fig. 11:**
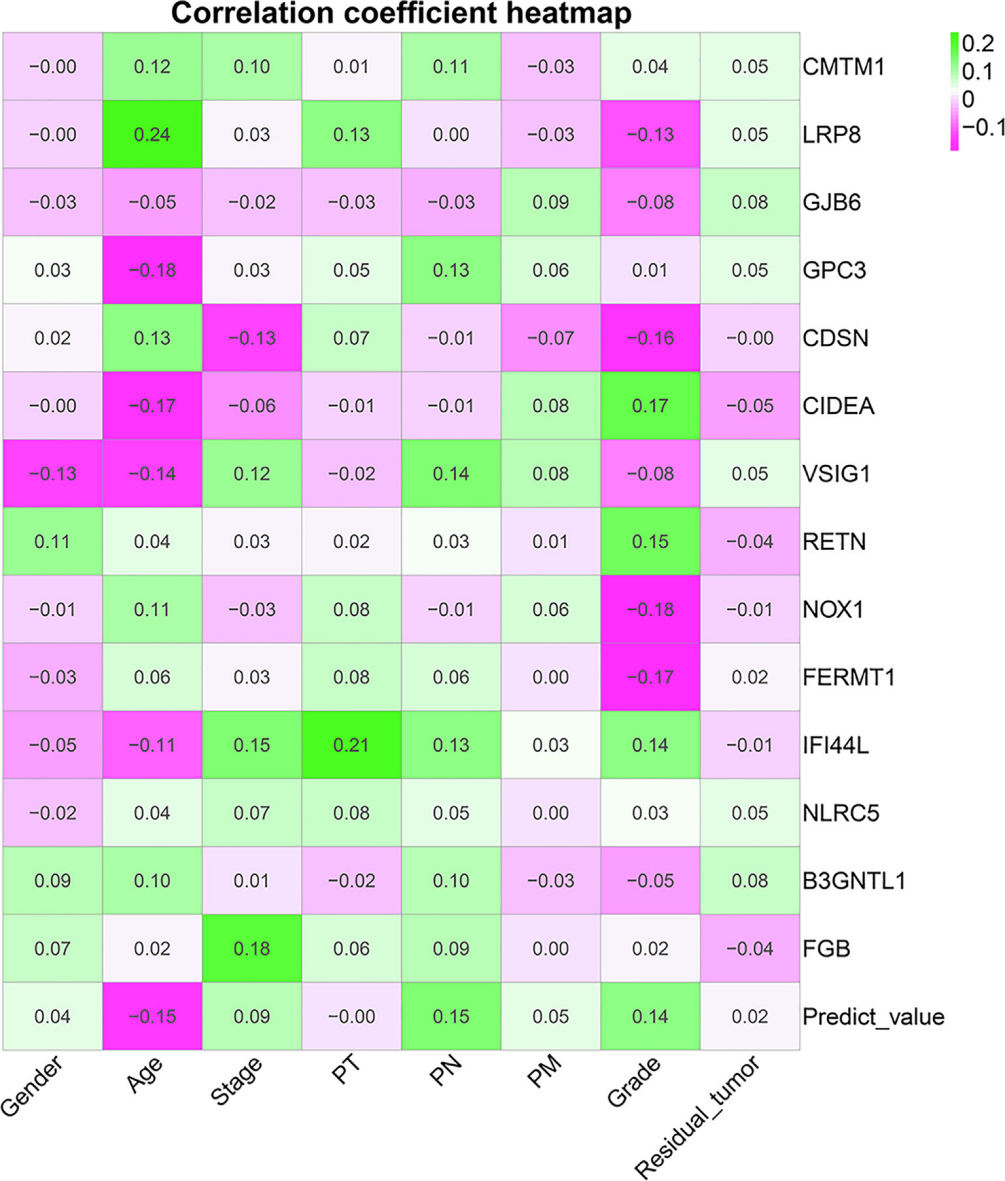
Clinical variable correlation coefficient heatmap.

Clinical correlation analyses displayed the correlation coefficient between clinical parameters and immune genes (Fig. 11). Supplementary Fig. 9 depicted correlation significance between clinical parameters and immune genes.

### 3.13 Tumor infiltrating immune cell correlation analyses

**Fig. 12:**
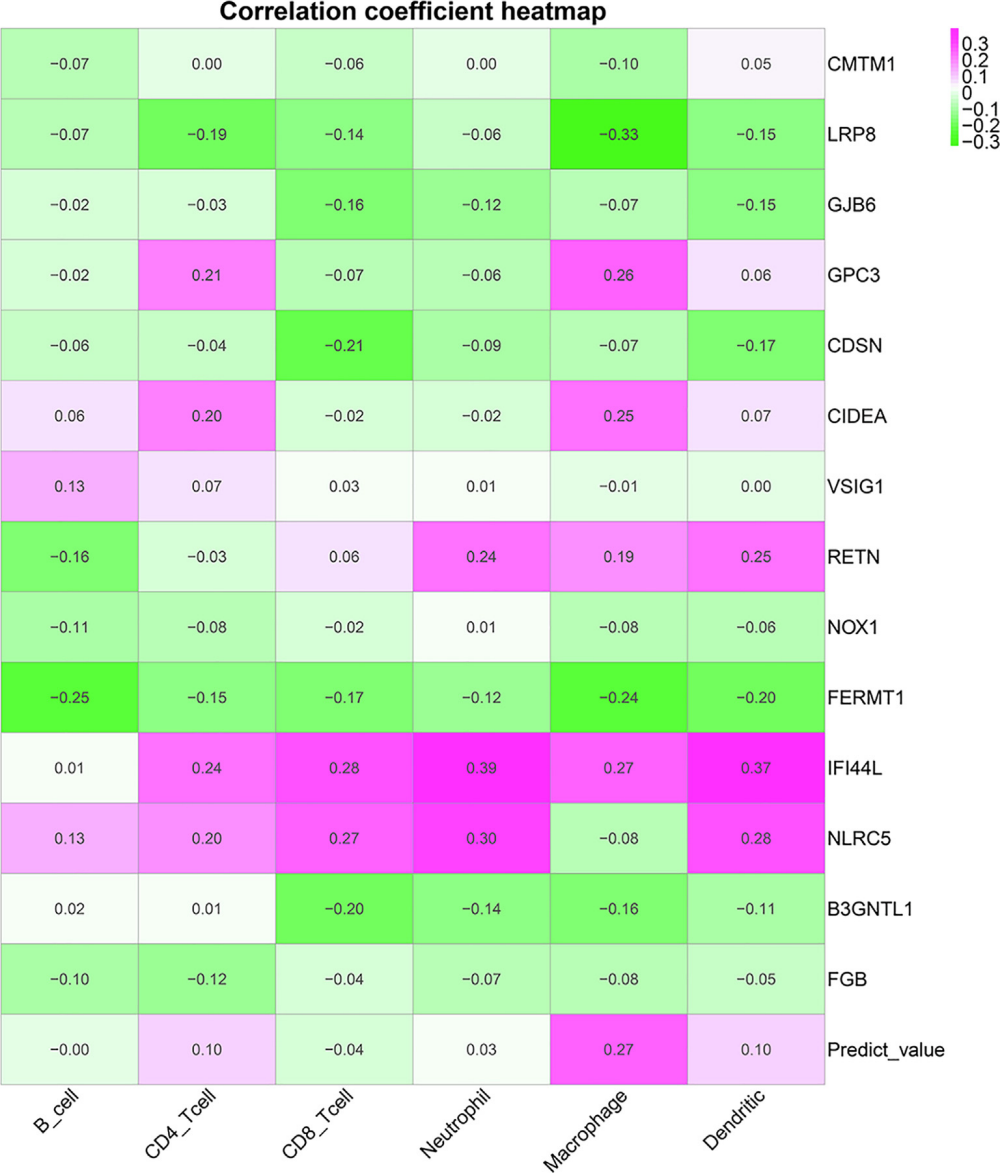
Immune gene correlation coefficient heatmap.

The original values of six tumor infiltrating immune cells (B_cell, CD4_Tcell, CD8_Tcell, Neutrophil, Macrophage, and Dendritic) were downloaded from Tumor IMmune Estimation Resource database. Fig. 12 showed the correlation coefficient between tumor infiltrating immune cells and immune genes. Supplementary Fig. 10 depicted correlation significance between tumor infiltrating immune cells and immune genes.

### 3.14 Tumor infiltrating immune cells

**Fig. 13:**
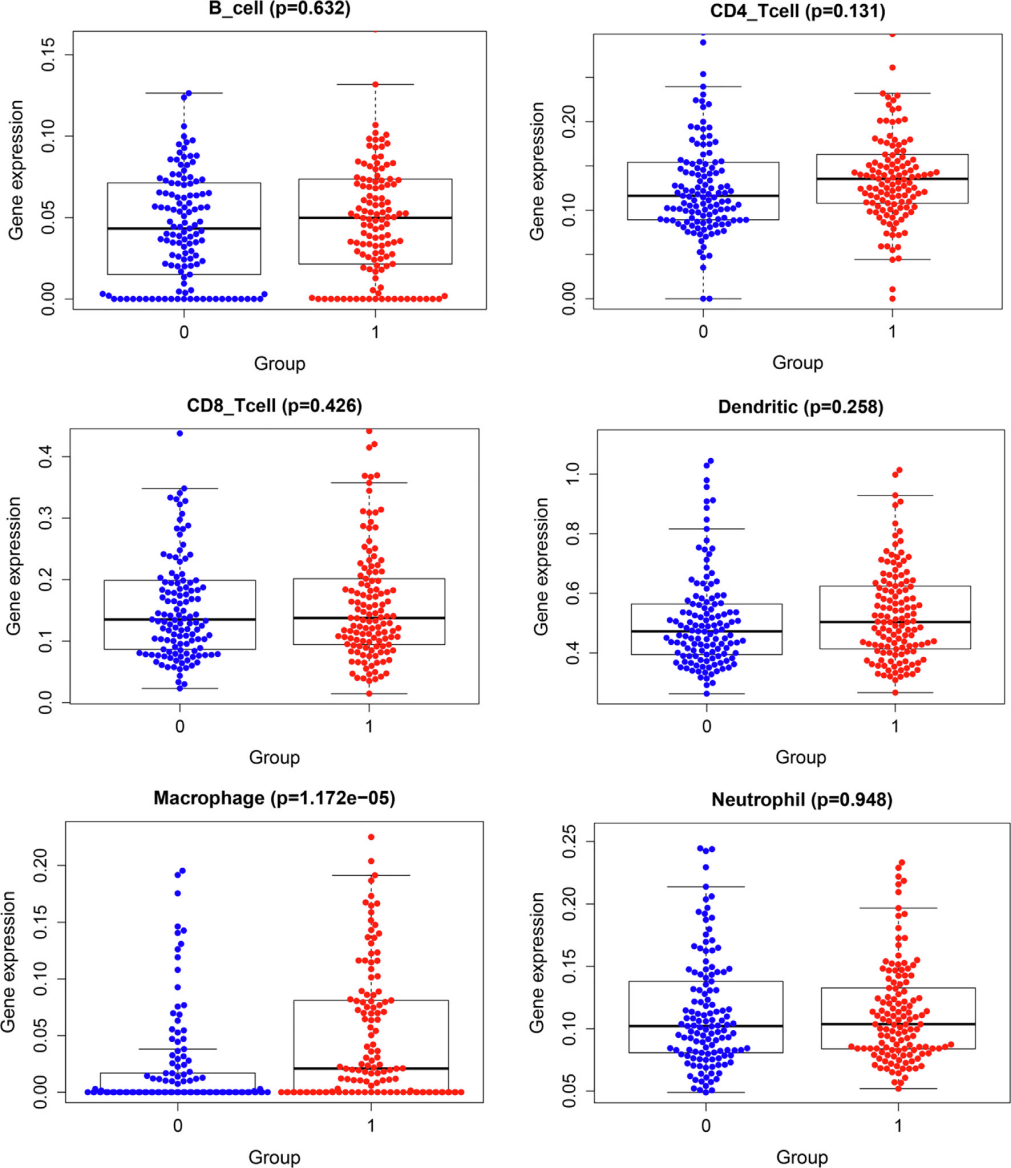
Expression of tumor immune infiltrating cells.

**Fig. 14:**
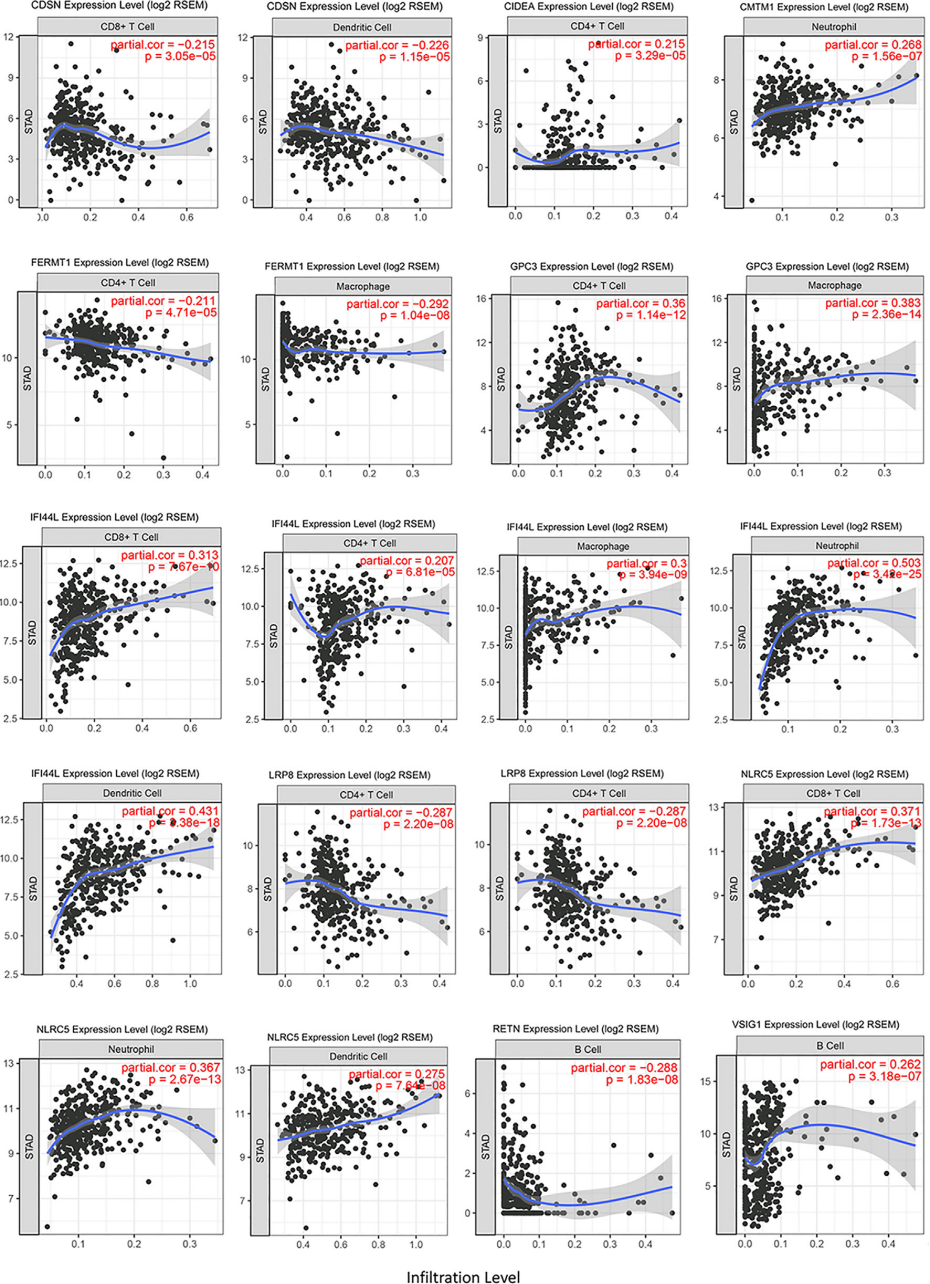
Scatter plot between tumor infiltrating immune cells and immune genes.

**Fig. 15:**
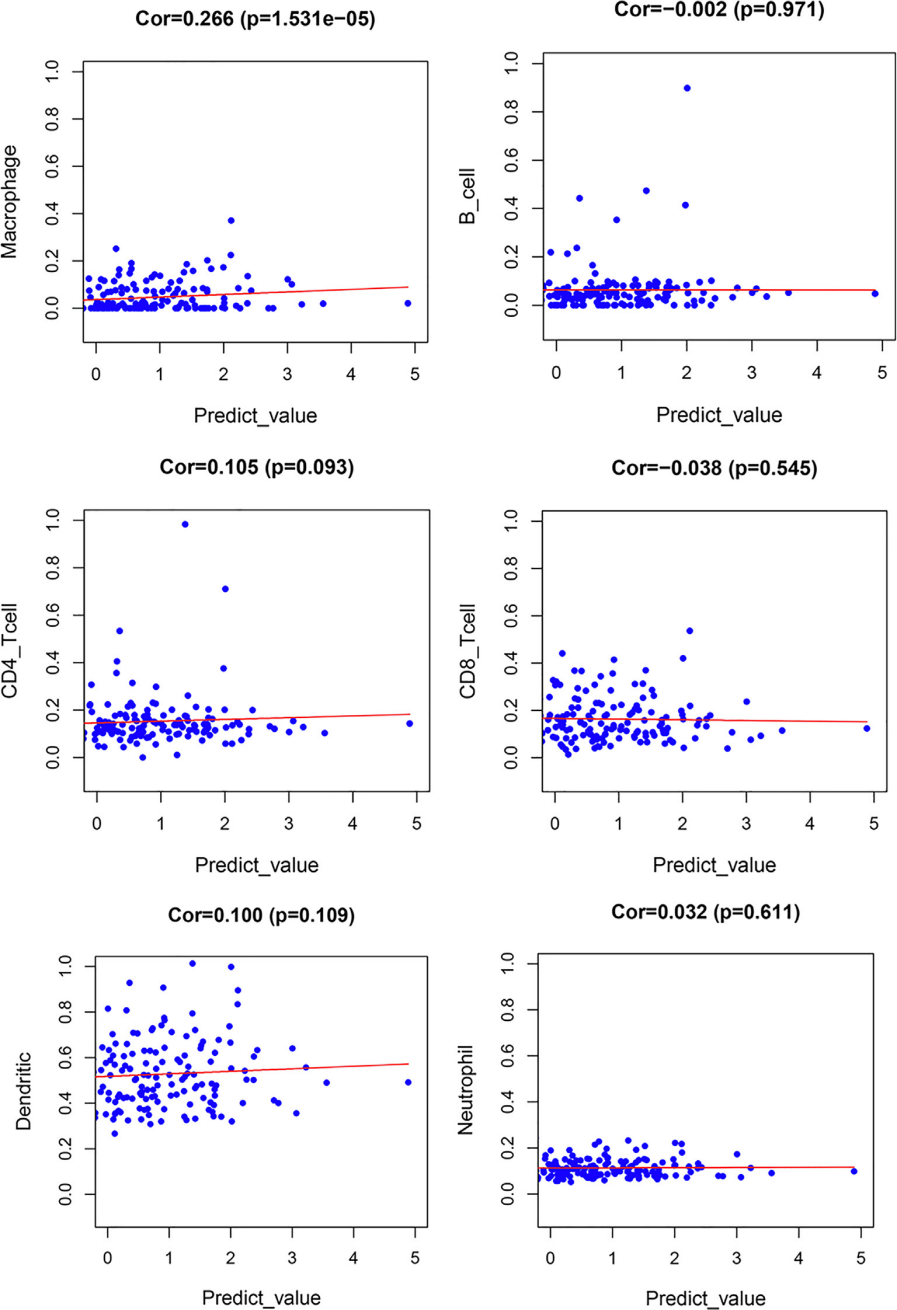
Scatter plot between tumor infiltrating immune cells and prognostic signature.

The median values were used to classify high-risk patients and low-risk patients. Expression of tumor infiltrating immune cells in patients with high risk score and low risk score was presented in Fig. 13. Scatter plots between tumor infiltrating immune cells and immune genes were shown in Fig. 14. Correlation analyses between tumor infiltrating immune cells and prognostic score were shown in Fig. 15.

### 3.15 Subgroup analyses among different races

Subgroup analyses demonstrated that there was no significant difference of immune gene prognostic signature among different races (Supplementary Fig. 11).

## 4 Discussion

The current study identified 14 immune genes closely related to the prognosis of gastric cancer. These immune genes may become valuable prognostic biomarkers and potential targets for tumor immunotherapy. The current study constructed a transcription factor regulatory network of immune genes, which may be helpful to understand the potential molecular regulatory mechanisms of tumorigenesis and progression. The current study developed and validated a prognostic signature for DFS of GC patients. In addition, we developed two novel artificial intelligence survival predictive tools to predict individual mortality risk. Additionally, the artificial intelligence survival predictive system could provide 95% confidence interval of predicted mortality and median survival time. These two artificial intelligence survival predictive tools were convenient in providing individualized mortality risk prediction with advantages of simple operation and intuitive results.

Previous studies have reported several prognostic models for predicting the prognosis of gastric cancer patients [16], [17]. However, these prognostic models can’t predict the mortality risk for an individual patient. In recent years, artificial intelligence algorithms, including Multi-task logistic regression algorithm, Cox survival regression algorithm, and random survival forest algorithm, have made great progress in survival prediction [21], [22], [23], [24], [25], [26]. With the supports of these advanced artificial intelligence algorithms, we have successfully established artificial intelligence survival predictive system to predict the mortality risk curve for an individual patient. Meanwhile, the current artificial intelligence survival predictive system could provide 95% confidence interval of predicted mortality and median survival time. Individual level survival prediction and median survival time prediction are the unique prediction ability of our artificial intelligence survival predictive system. In the current study, we creatively applied three artificial intelligence algorithms for predicting the individual mortality risk of cancer patients, providing a feasible idea and valuable reference for the future survival prediction studies.

The current research searched TISIDB databases to explore the biological process of these immune genes (http://cis.hku.hk/TISIDB/index.php). The major biological process of DFFA Like Effector A (CIDEA) is DNA catabolic process, endonucleolytic, temperature homeostasis, and negative regulation of cytokine production. The major biological process of V-set and immunoglobulin domain containing 1 (VSIG1) is tissue homeostasis, epithelial cell development, and epithelial cell morphogenesis. The major biological process of fermitin family member 1 (FERMT1) is ameboidal-type cell migration, establishment or maintenance of cell polarity, and epithelial cell migration. The major biological process of resistin (RETN) is positive regulation of collagen metabolic process, aging and regulation of collagen metabolic process. The major biological process of NLR family, CARD domain containing 5 (NLRC5) is negative regulation of immune system process, response to virus, and positive regulation of cytokine-mediated signaling pathway. The major biological process of gap junction protein, beta 6, 30 kDa (GJB6) is cellular glucose homeostasis, response to molecule of bacterial origin, and aging. The major biological process of glypican 3 (GPC3) is retinoid metabolic process, morphogenesis of a polarized epithelium, and ossification. The major biological process of interferon-induced protein 44-like (IFI44L) is response to virus, defense response to virus, and defense response to other organism. The major biological process of low density lipoprotein receptor-related protein 8 (LRP8) is regulation of cell morphogenesis involved in differentiation, retinoid metabolic process, and isoprenoid metabolic process. The major biological process of fibrinogen beta chain (FGB) is extrinsic apoptotic signaling pathway via death domain receptors, vascular process in circulatory system, and adaptive immune response. The major biological process of NADPH oxidase 1 (NOX1) is oxidoreduction coenzyme metabolic process, angiogenesis, and response to oxidative stress. The major biological process of corneodesmosin (CDSN) is keratinocyte differentiation, epidermis development, and epidermal cell differentiation.

The current study identified several valuable prognosis-related biomarkers, which might be potential candidates in targeted treatment. Huang Y et al. reported that methylation level of Cell Death Inducing CIDEA was related with tumor microsatellite instability [40]. Cell proliferation was mediated by NADPH Oxidase 1 (Nox1) expression in colon carcinoma cell lines [41]. High expression of Nox1 in colon cancer accelerated the tumor growth and inhibition of Nox1 might become a new therapeutic strategy for colorectal cancer treatment [42]. Low expression of Interferon Induced Protein 44 Like (IFI44L) impaired antiviral state induced by IFN and might be potential candidate for reduction of virus replication [43]. Glypican 3 (GPC3) was potential immune target for hepatocellular carcinoma through fusing to alpha epitope of HBsAg [44]. GPC3-S-Fab could kill GPC3 positive hepatocellular carcinoma cells through natural killer cells [45]. NLR Family CARD Domain Containing 5 (NLRC5) had a weak moderate effect for modulating CD8 + T-cell responses in mice small intestine with rotavirus infection [46]. NLRC5 could mediate proliferation, migration and invasion of renal cell carcinoma through wnt/beta-catenin signaling pathway [47]. Previous studies indicated potential effects of immune genes in molecular biological regulatory mechanisms and pathways of tumorigenesis and progression. The current study constructed a transcription factor regulatory network of immune genes. This regulatory network was helpful to reveal the potential role of immune genes in tumorigenesis and progression.

Tumor infiltrating macrophages could express interleukin 25, which was significantly related to the prognosis of gastric cancer after radical resection [48]. Macrophages could enhance the invasiveness of gastric cancer cells by enhancing the transforming growth factor beta / bone morphogenetic protein pathway [49]. High expression of CD8 + T cells was associated with prognosis and lymph node metastasis of gastric cancer [50]. High regulatory T cells to CD8 + T cells ratio was significantly correlated with poor prognosis of gastric cancer [51]. High infiltration of CD8 + T cell increased programmed death ligand 1 and decreased survival rate [52]. Tumor antigen could stimulate CD8 + T cells [53], [54]. Neutrophils could inhibit the anti-tumor ability of dendritic cells [55]. Pro-tumoral neutrophils could up-regulate immunosuppressive dendritic cells [56]. Dendritic cell infiltration plays an important role in the initiation of primary anti-tumor immune response [57]. Neutrophils could inhibit immune response and accelerate the progress of gastric tumors via GM-CSF-PD-L1 pathway [58].

Advantages of current study: The current research developed artificial intelligence predictive tools for GC patients based on three artificial intelligence algorithms. Artificial intelligence survival predictive system was convenient to predict individualized mortality risk with visual illustration and numerical presentation. The artificial intelligence predictive tools can provide more accurate individual prognostic information and are more suitable to meet the needs of individualized treatment and precision medicine. In order to provide more reliable prognostic information for individual patient, three individual mortality risk predictive curves were presented based on different artificial intelligence algorithms. The current artificial intelligence survival predictive system could provide 95% confidence interval of predicted mortality and median survival time.

Shortcomings of current study: First, the current research explored clinical significance of immune genes in tumorigenesis and progression based on datasets from public databases. However the conclusions have not yet been verified by researchers' own research data. Second, sample size of the current research is relatively small, weakening the credibility of research conclusions to a certain extent. Third, some patients with gastric cancer have comorbidities and the other cancers. The current study did not consider the impacts of comorbidities and the other cancers on the individual mortality curve. Fourth, due to the lack of efficacy indicators of radiotherapy and chemotherapy, our predictive system can’t predict the efficacy of different treatment regimens for cancer patients. Fifth, overall survival is a valuable outcome for prognostic evaluation of gastric cancer. However, due to the lack of effective clinical dataset, the current study did not explore and establish the prognostic model for gastric cancer patients by using overall survival as final outcome. Prospective basic researches are helpful to further explore the potential role of immune genes in molecular biological regulatory mechanism of tumorigenesis and progression. Third, due to the complexity of artificial intelligence algorithms, the calculation process could not be displayed by simple formula, blocking the application of artificial intelligence algorithms in the field of tumor prognosis to a certain extent.

## 5 Conclusion

The current study constructed a transcription factor regulatory network and developed two artificial intelligence survival prediction tools (https://zhangzhiqiao7.shinyapps.io/Smart_Cancer_Survival_Predictive_System_15_GC_D1006/ and https://zhangzhiqiao7.shinyapps.io/Gene_Survival_Subgroup_Analysis_15_GC_D1006/) for disease free survival of gastric cancer patients. These artificial intelligence survival prediction tools are helpful to predict individual mortality risk and provide valuable prognostic information for individualized treatment decision.

## 6 Ethics approval and consent to participate

The studies in TCGA database and GEO database have received ethical approval from ethics committees of their respective research institutes. These studies obtained informed consent from patients before admission. The current study is a second study based on public datasets from TCGA database and GEO database. Details of all patients in public datasets have been anonymously processed and therefore the current research does not involve patients' privacy information. The current study was performed according to public database policy and declaration of Helsinki. TCGA database and GEO database allows researchers to use public datasets for scientific purposes. Ethical approval of this study was waived in accordance with the recommendations of Management Measures for Ethical Review of Clinical Research, Ethics Committees of Shunde Hospital, Southern Medical University because the current study was a retrospective study based on public datasets. Therefore ethical approval and informed consent were not required for the current study.

## 7 Consent for publication

All authors approved the publication.

## 8 Availability of data and materials

The study data is available at: https://zhangzhiqiao7.shinyapps.io/Gene_Survival_Subgroup_Analysis_15_GC_D1006/.

## Funding

The current research was funded by Medical Science and Technology Foundation of Guangdong Province (B2018237). Foshan Science and Technology Bureau (2020001004584).

## CRediT authorship contribution statement

**Zhiqiao Zhang:** Conceptualization, Methodology, Resources, Investigation, Data curation, Formal analysis, Validation, Software, Project administration, Supervision, Visualization, Funding acquisition. **Tingshan He:** Conceptualization, Methodology, Resources, Investigation, Data curation, Formal analysis, Validation, Software, Project administration, Supervision. **Liwen Huang:** Conceptualization, Methodology, Resources, Investigation, Data curation, Formal analysis, Validation, Software, Project administration, Supervision. **Jing Li:** Conceptualization, Methodology, Resources, Investigation, Data curation, Formal analysis, Validation, Software, Project administration, Supervision. **Peng Wang:** Conceptualization, Methodology, Resources, Investigation, Data curation, Formal analysis, Validation, Software, Project administration, Supervision, Visualization.

## Declaration of Competing Interest

The authors declare that they have no known competing financial interests or personal relationships that could have appeared to influence the work reported in this paper.
