# Supplementary material for: Immune gene prognostic signature for disease free survival of gastric cancer: Translational research of an artificial intelligence survival predictive system
Source: Comput Struct Biotechnol J. 2021 Apr 12;19:2329–46. doi: 10.1016/j.csbj.2021.04.025 (PMC8111455; doi:10.1016/j.csbj.2021.04.025)
Supplement: Supplementary data 1 [file mmc1.pdf]

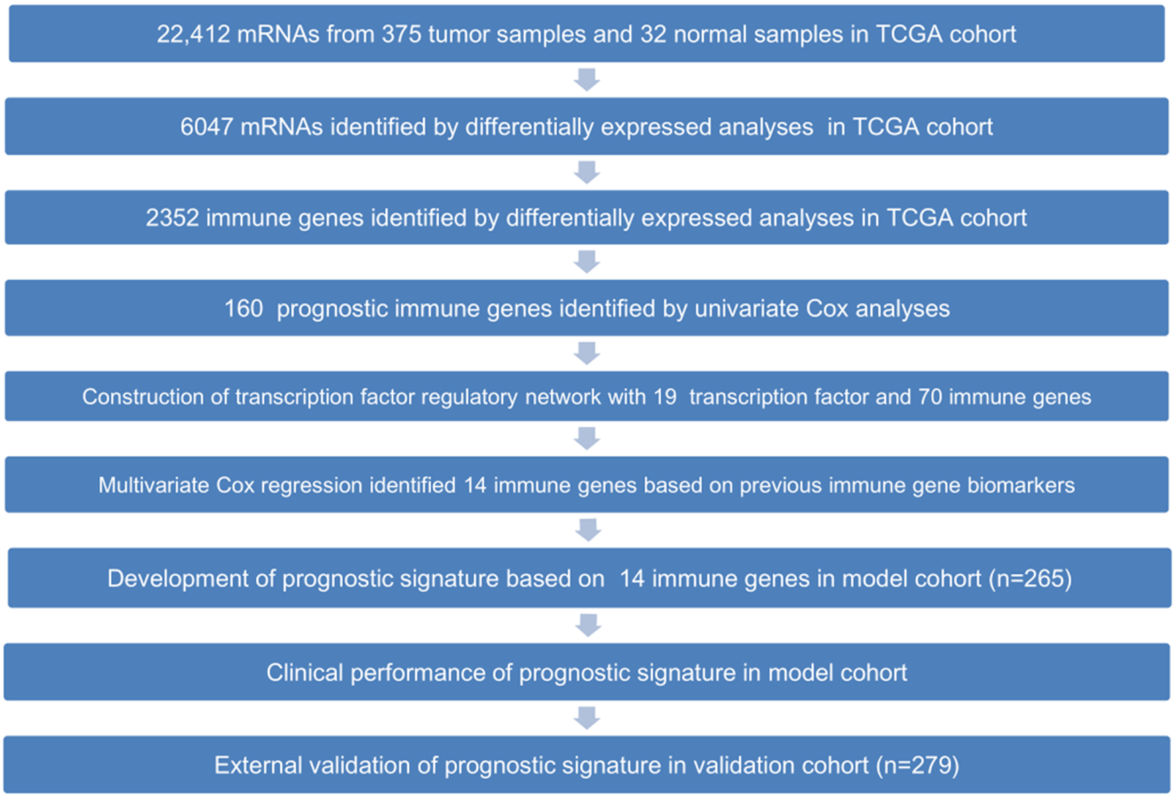

Supplementary Figure 1. Flow chart in current study

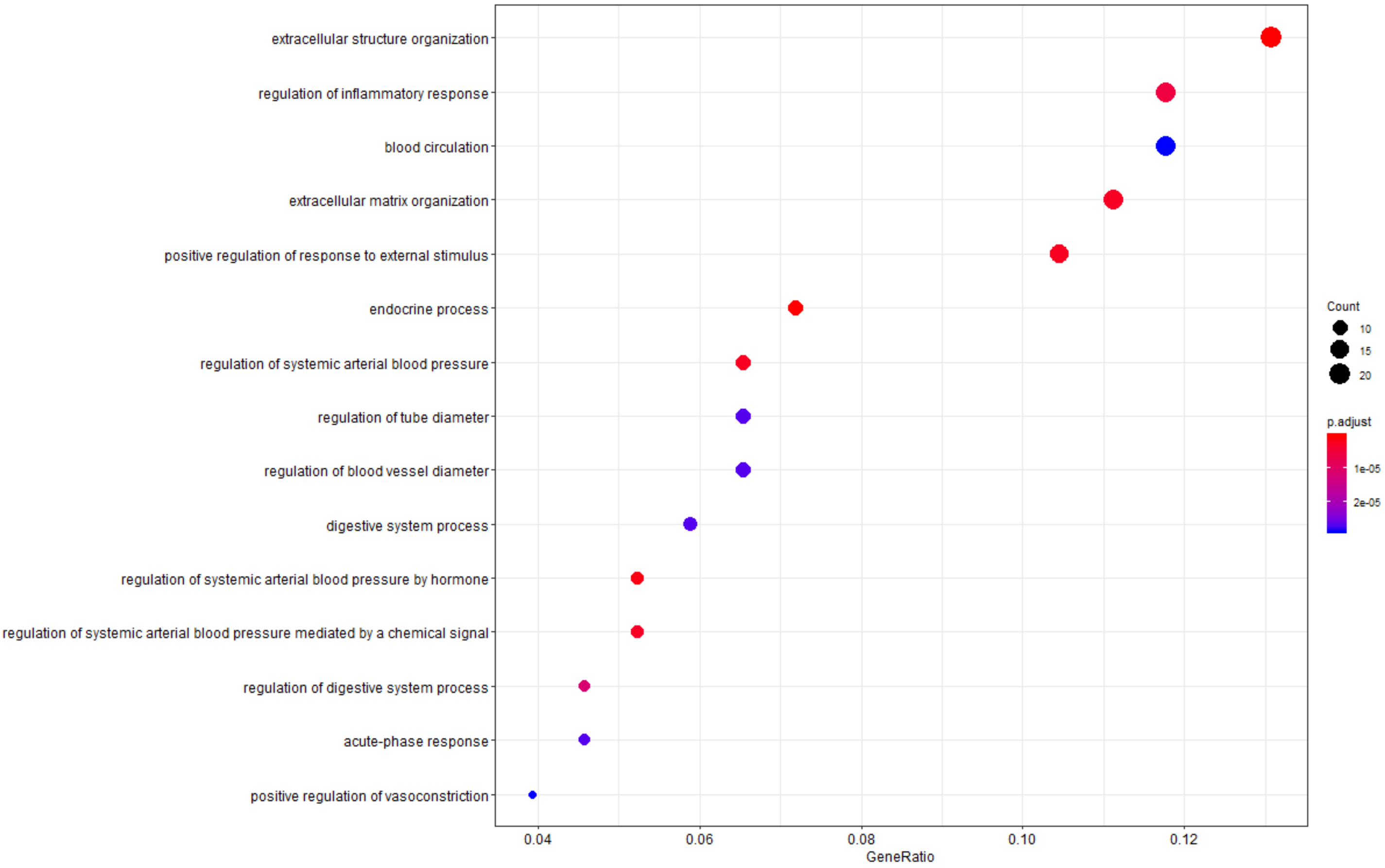

Supplementary Figure 2. Bubble chart of prognostic genes

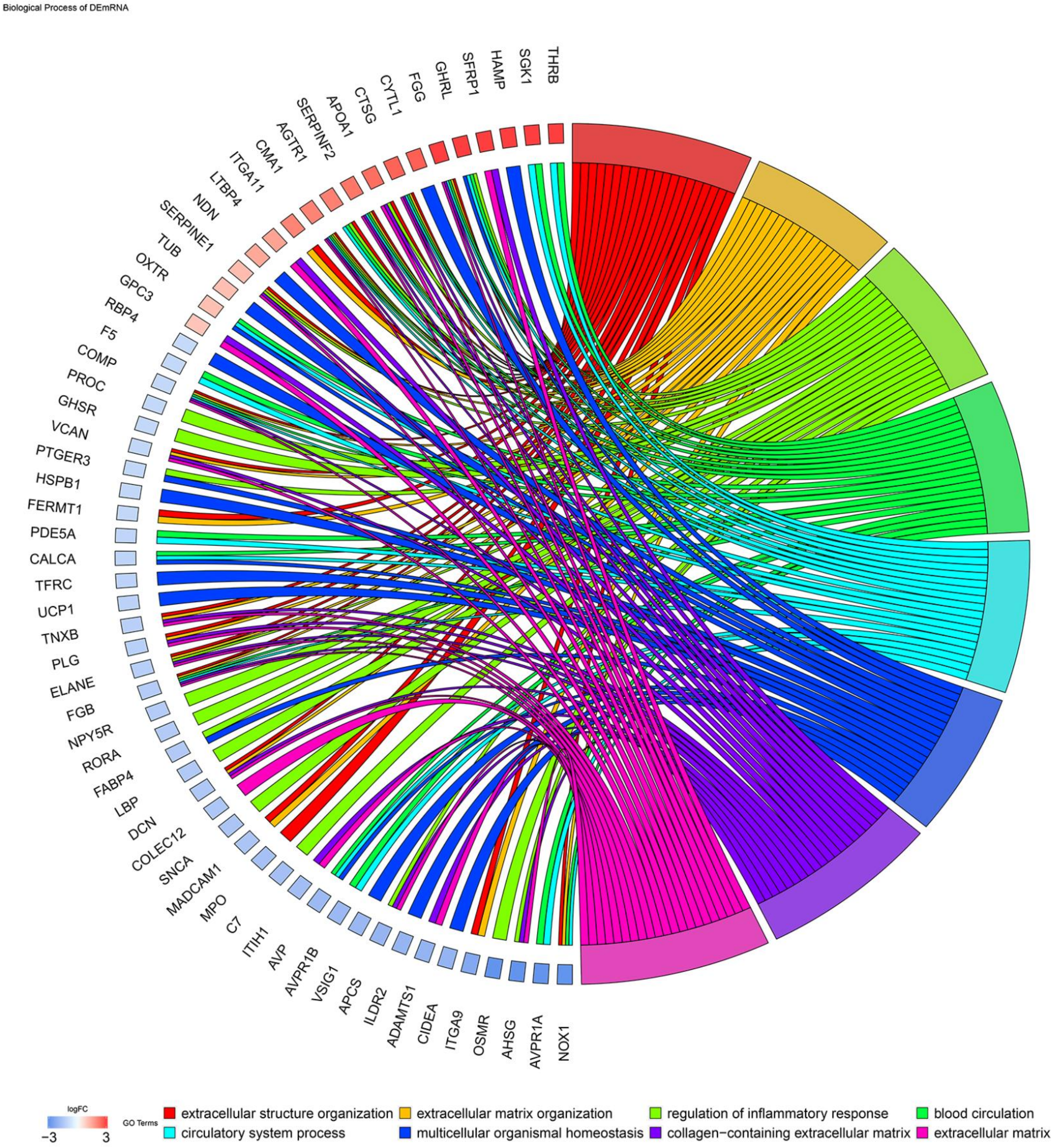

Supplementary Figure 3. Chord chart of prognostic genes

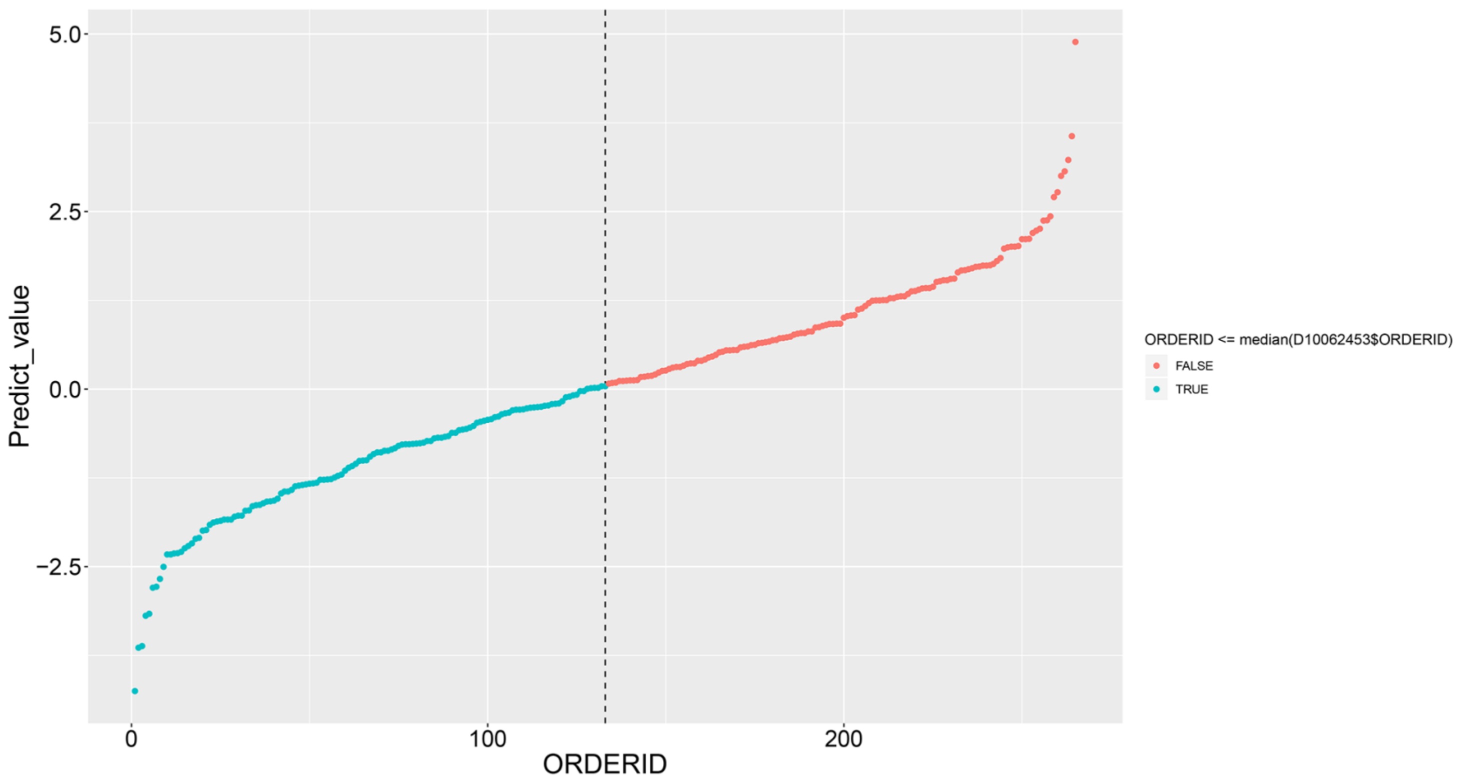

Supplementary Figure 4. Predictive value distribution chart

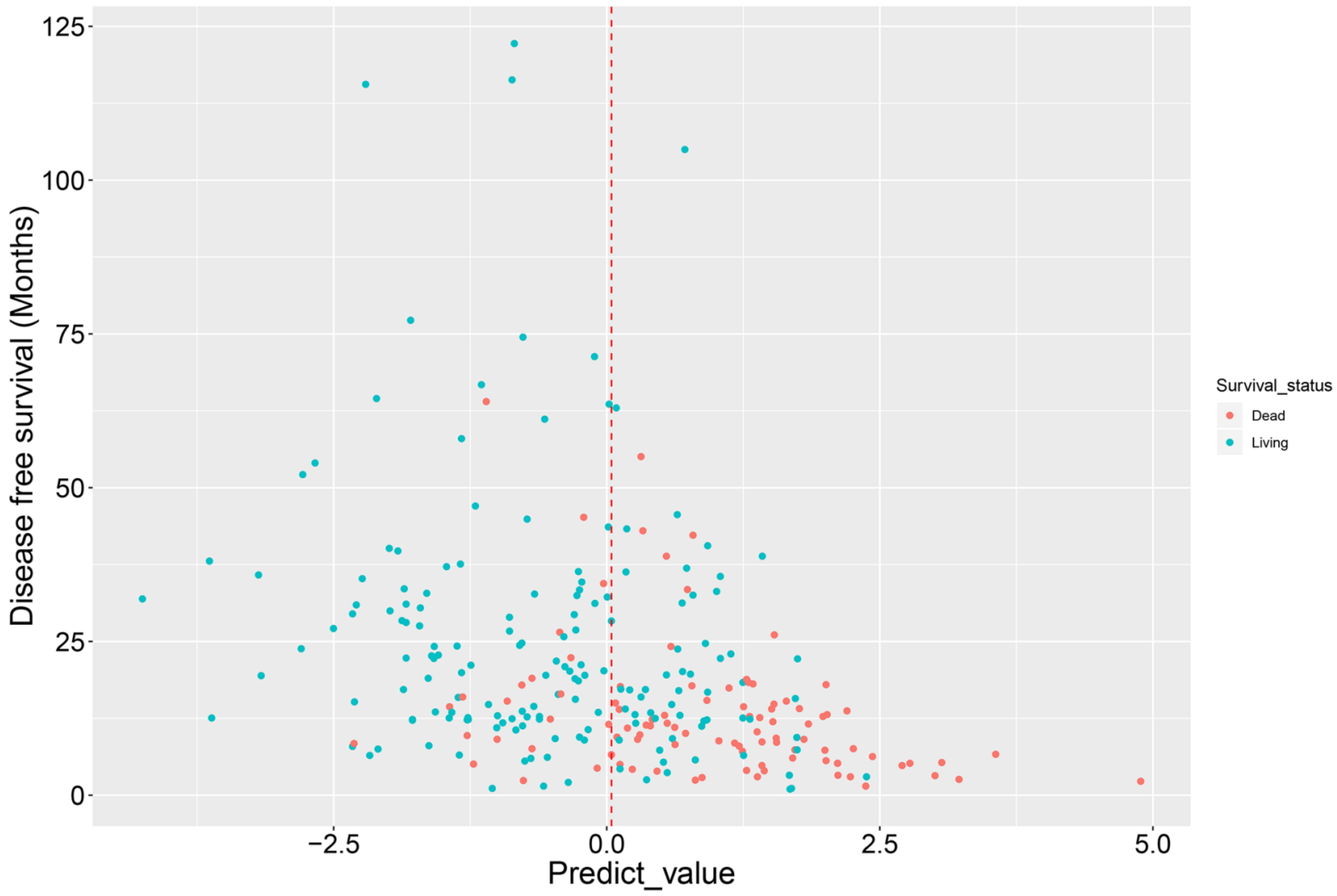

Supplementary Figure 5. Survival status scatter plot

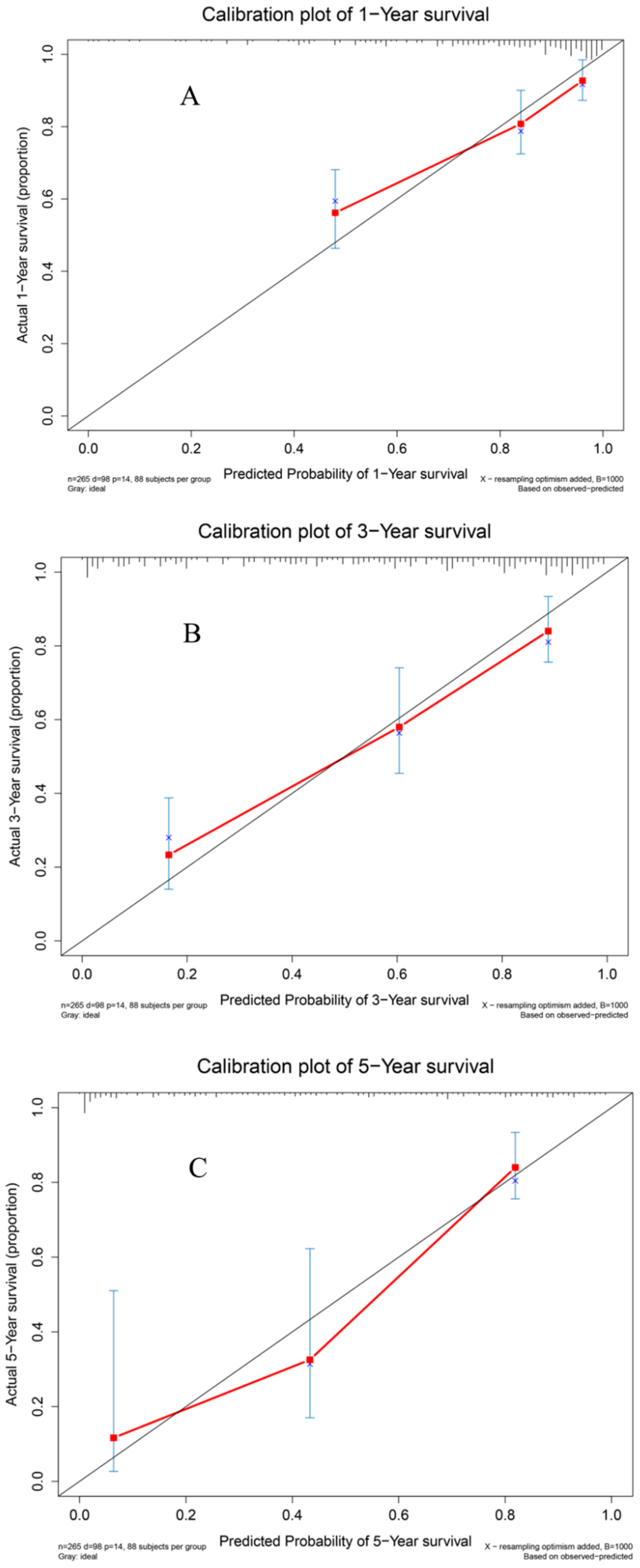

Supplementary Figure 6. Calibration curves for model cohort: (A) Calibration curve for 1-year survival; (B) Calibration curve for 3-year survival; (C) Calibration curve for 5-year survival

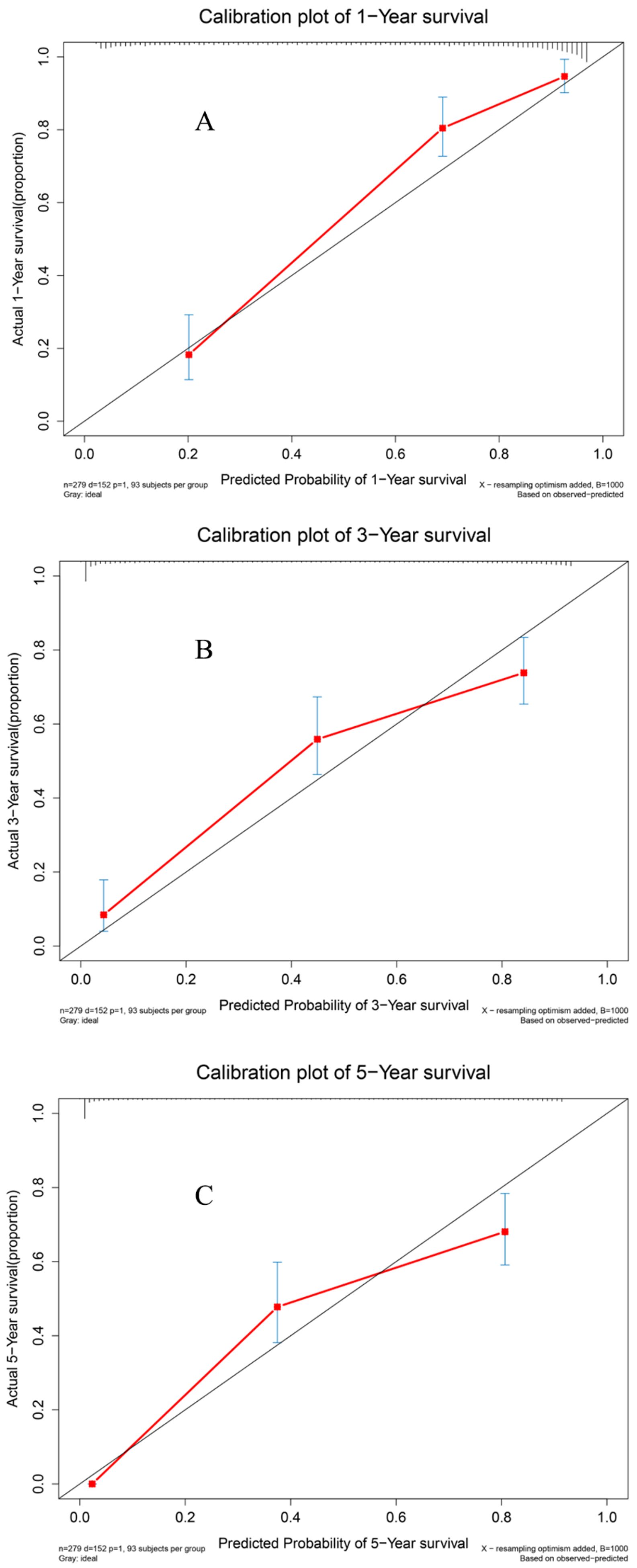

Supplementary Figure 7. Calibration curves for validation cohort: (A) Calibration curve for 1-year survival; (B) Calibration curve for 3-year survival; (C) Calibration curve for 5-year survival

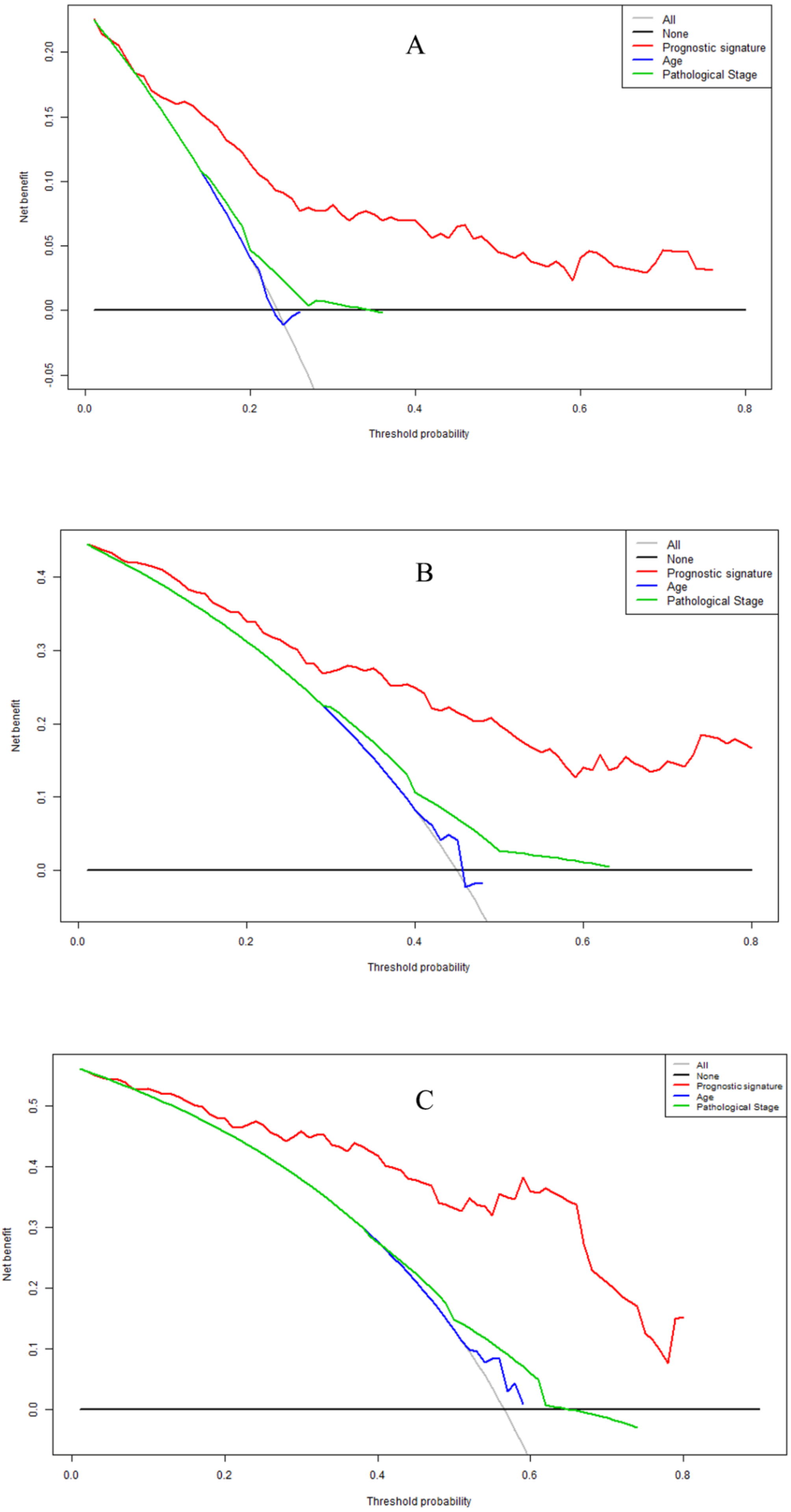

Supplementary Figure 8. Decision curve chart：(A) Decision curve chart for 1-year survival; (B) Decision curve chart for 3-year survival; (C) Decision curve chart for 5-year survival

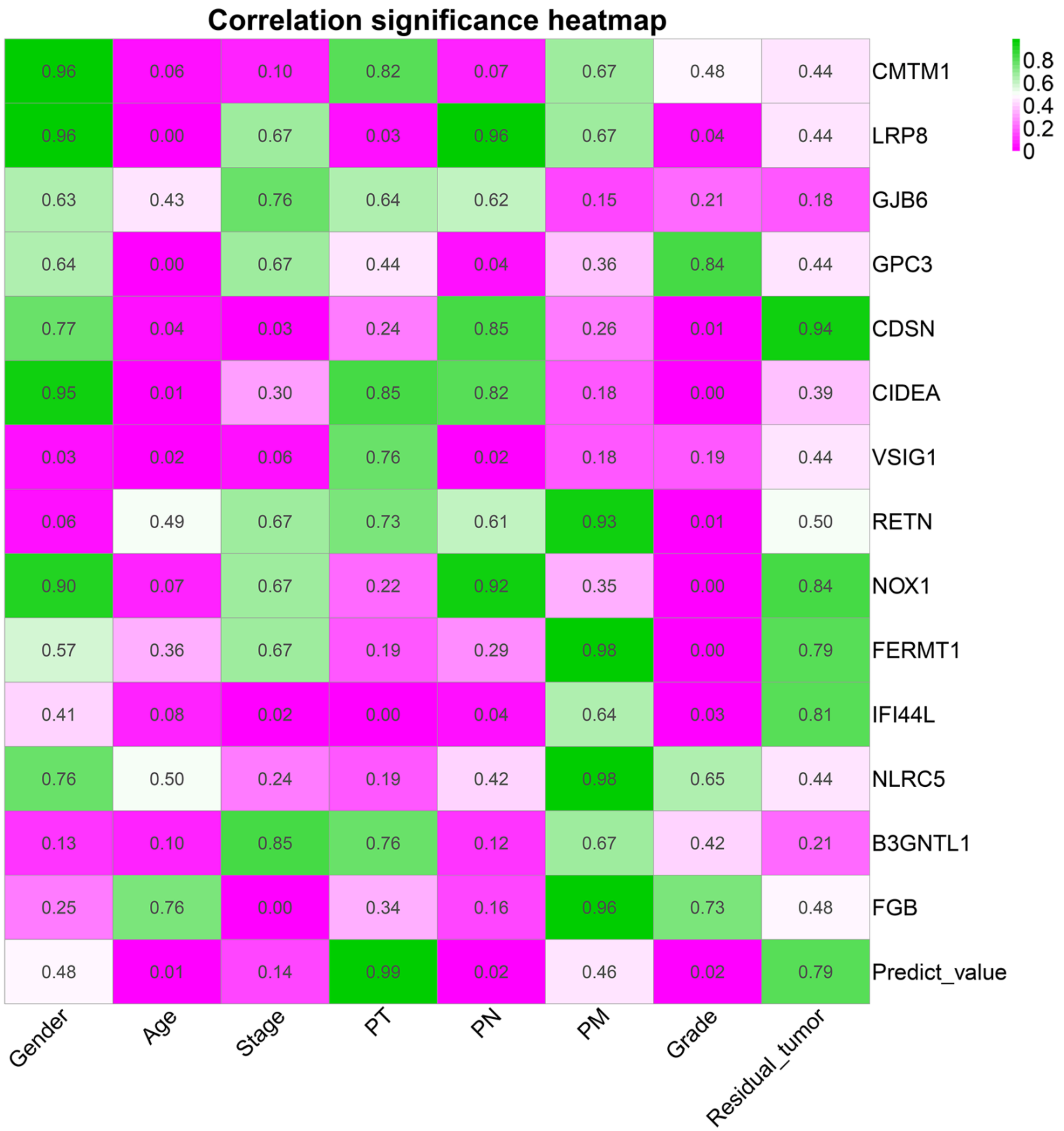

Supplementary Figure 9.Clinical variable correlation significance heatmap

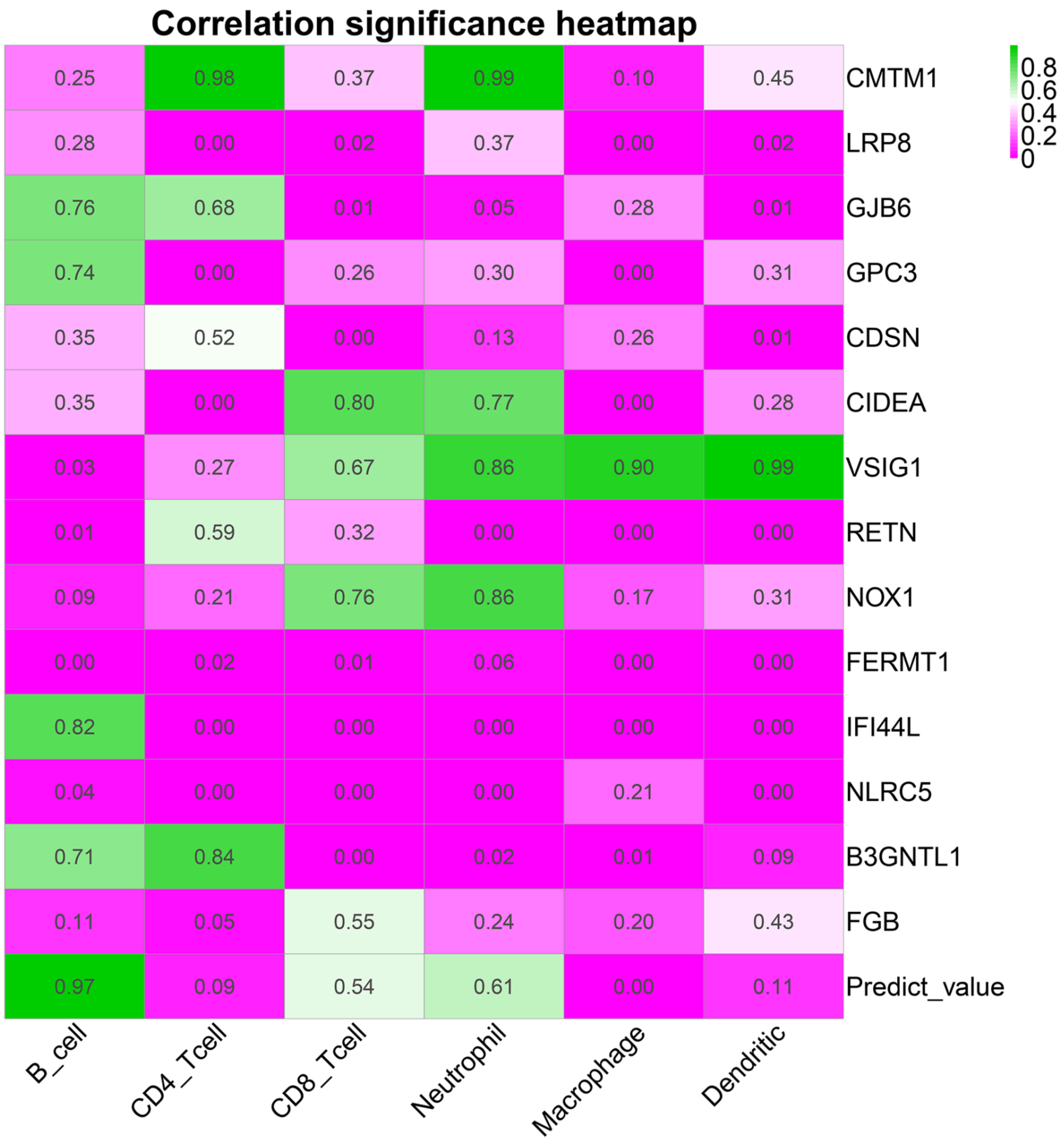

Supplementary Figure 10.Immune gene correlation significance heatmap

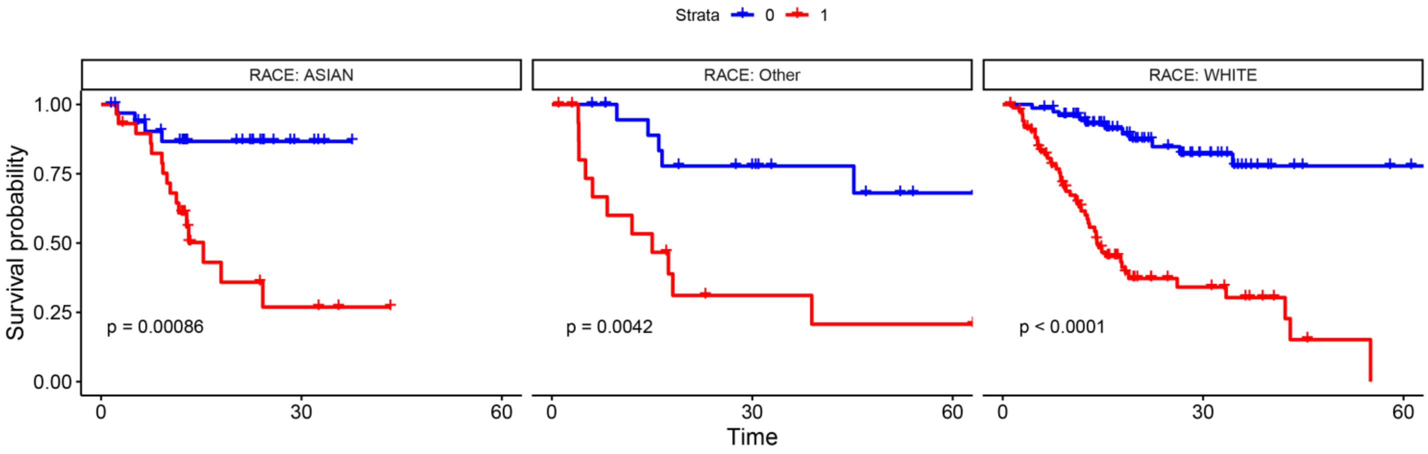

Supplementary Figure 11. Subgroup analyses among different races
